# A Coding SNP in *GmPM30* Enhances Soybean Salinity Tolerance and Yield through the GmLEA1‐GmPM30‐GmLEC1 Module

**DOI:** 10.1002/advs.202509391

**Published:** 2025-10-06

**Authors:** Shiyu Huang, Yuhan Xia, Jingting Yang, Yujun Si, Xue Chen, Hao Zhang, Tianshi Liu, Wenyu Zheng, Xin Chen, Zhongjuan Zhao, Xiaojian Zheng, Qing Lu, Shuo Li, Fengning Xiang

**Affiliations:** ^1^ The Key Laboratory of Plant Development and Environmental Adaptation Biology Ministry of Education Shandong Key Laboratory of Precision Molecular Crop Design and Breeding School of Life Sciences, Shandong University Qingdao 266237 China; ^2^ Weifang Academy of Agricultural Sciences Weifang 261071 China

**Keywords:** natural allelic variation, salt tolerance, seed maturation protein, soybean, yield

## Abstract

Salt stress limits soybean quality and yield. Despite the genetic validation of many salinity tolerance genes, the roles and regulatory mechanisms of their natural variations in population‐level salt tolerance remain unclear. This study identifies seed maturation protein PM30 (GmPM30), a *late embryogenesis abundant (LEA)* gene, as enhancing soybean salt tolerance. A significant T/C nonsynonymous polymorphism in the coding region of *GmPM30* confers haplotype *HapT* with greater salt tolerance than *HapC* via stronger GmLEA1‐GmPM30‐GmLEC1 (Lectin) interactions, reducing ion leakage, malondialdehyde (MDA) content, and hydrogen peroxide (H_2_O_2_) accumulation under salt stress. RNA‐seq demonstrates that *GmPM30‐HapT* activates more extensive and robust stress response pathways than *HapC*. Evolutionary analyses reveal artificial selection of the *GmLEA1‐GmPM30‐GmLEC1* module during soybean domestication and breeding, with *GmPM30* being geographically adapted to high‐latitude regions with greater saline‐alkaline stress. Pyramiding lines with elite alleles (*GmLEA1‐Hap3‐GmPM30‐HapT‐GmLEC1‐Hap3*) boosts grain yield on saline soil. An efficient marker developed for *GmPM30‐HapT* enables marker‐assisted selection (MAS) breeding, and haplotype hybrids with successful *GmPM30‐HapT* integration exhibit improved yield‐related traits in saline farmlands. This study establishes a novel workflow linking evolutionary genomics, molecular mechanisms, and breeding applications through the *GmLEA1‐GmPM30‐GmLEC1* module, providing a replicable blueprint for rapid crop improvement by using natural selection strategies.

## Graphical abstract

A natural variation in soybean *PM30*, *GmPM30‐HapT*, confers greater salt tolerance than *GmPM30‐HapC* via stronger GmLEA1‐GmPM30‐GmLEC1 interactions. Pyramiding their elite haplotypes yields additive gains in salt tolerance and yield, establishing a novel workflow linking evolutionary genomics, molecular mechanisms, and breeding applications via the module to guide soybean salt tolerance research using natural selection.

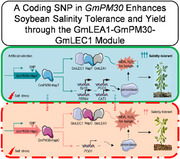

## 1 Introduction

Soybean (*Glycine max* (L.) Merr.) is one of the most economically important crops. Indeed, its abundant seed oil and high‐quality vegetable protein serve as major sources of human and animal nutrition.^[^ ^1^ ^]^ Cultivated soybean was domesticated from the wild soybean *Glycine soja* (Sieb. & Zucc) in China ≈5000 years ago.^[^ ^2^ ^]^ However, during long‐term domestication, artificial selection has resulted in the loss of genetic diversity in currently cultivated soybeans, leading to diminished ability to resist various stresses, compared to *G. soja* (Sieb. & Zucc). High salinity is a key limiting factor for crop growth and production, which is worsening due to the continuous expansion of saline arable land.^[^ ^3^ ^]^ Therefore, it is of great importance to increase the salinity tolerance of crops and breed soybean varieties with high yield and greater stress tolerance to make full use of such saline soils. Moreover, the identification and exploitation of soybean salt tolerance genes have important theoretical and practical significance for molecular breeding of salt tolerance.

In recent years, many genes related to salinity stress tolerance have been identified in soybean by reverse genetics, such as *Na^+^/H^+^ Antiporter 1* (*GmNHX1*),^[^ ^4^ ^]^ *Cation/proton antiporter 1* (*GmCAX1*),^[^ ^5^ ^]^ *Chloride channel 1* (*GmCLC1*),^[^ ^6^ ^]^ *AKT1‐type K^+^ channel* (*GmAKT1*),^[^ ^7^ ^]^ and transcription factor genes encoding members from the NAM ATAF CUC (NAC), MYB, basic leucine zipper (bZIP), and WRKY families.^[^ ^8^, ^9^, ^10^, ^11^, ^12^, ^13^, ^14^ ^]^ However, studies on natural variation of salt tolerance genes remain limited. Natural variation, domestication, and artificial/geographical selection of functional genes are also crucial for enhancing the adaptation, resistance, yield, and quality of crops.^[^ ^15^ ^]^ In soybeans, only a few quantitative trait loci (QTLs) and polymorphisms related to salinity tolerance have been identified. For example, the major salt tolerance soybean gene *GmSALT3*, encoding a cation/H^+^ antiporter, was identified using a genome‐wide association study^[^ ^16^ ^]^ and QTL mapping, respectively.^[^ ^17^ ^]^ In addition, a 7‐bp deletion in the *EARLY RESPONSE TO DEHYDRATION 15 B* (*GsERD15B*) promoter enhanced the salt tolerance of soybean.^[^ ^18^ ^]^ Loss of function in the flowering time–related protein E2 pleiotropically modulated soybean salt tolerance and early maturity.^[^ ^19^ ^]^ Although many genes have been genetically validated, few studies have made full use of population genetics to analyze genetic variation, identify elite alleles, and apply them to molecular breeding for natural populations.

Late Embryogenesis Abundant (LEA) proteins are mostly hydrophilic, thermally stable intrinsically disordered proteins (IDPs) that can maintain membrane integrity under abiotic stress. LEAs can alleviate oxidative damage in stressed plants by scavenging hydroxyl and peroxy radicals or binding metals.^[^ ^20^ ^]^ In addition, LEAs can also bind to misfolded proteins through molecular chaperones and restore their original conformation and biological activity.^[^ ^21^ ^]^ Thus, the production of highly hydrophilic LEAs is a common response mechanism to salinity stress in many plant species.^[^ ^22^, ^23^, ^24^, ^25^ ^]^ There have been several studies reporting that LEA proteins enhance salt tolerance in *Arabidopsis thaliana*.^[^ ^26^, ^27^, ^28^ ^]^ The GmLEA2‐1 (cultivar soybean) and GsPM30 (wild soybean) proteins were previously shown to enhance salinity and drought tolerance when overexpressed in *Arabidopsis thaliana*.^[^ ^29^, ^30^ ^]^ As a Group 3 LEA protein, soybean PM18 protein shows enhanced yeast salt tolerance when overexpressed in the yeast heterologous expression system.^[^ ^31^ ^]^ Previous studies have revealed that LEA proteins regulate plant stress responses through multiple mechanisms. Transcriptionally, *GmCOL1a* binds to *GmLEA* promoters to upregulate gene expression.^[^ ^30^ ^]^ Additionally, LEA proteins interact with kinases, potentially modulating their own activity.^[^ ^32^ ^]^ However, functional validation and molecular mechanism analysis of salt tolerance in stable transgenic soybeans, as well as evolutionary and population genetic analyses of genes encoding LEA proteins in soybeans, remain unreported.

Based on our previous microarray transcriptome analysis of soybean under salinity and control conditions, we cloned the soybean seed maturation protein PM30 (*GmPM30*; Gene ID: 547996; *Glyma. 13G363300*), a *LEA* gene whose expression was most strongly induced by salinity stress.^[^ ^33^ ^]^ We analyzed the role of *GmPM30* in mediating salt tolerance and grain yield traits. By integrating evolutionary genomics, molecular mechanisms, and functional genomics, we revealed that distinct *GmPM30* haplotypes differentially regulated the interactions of GmPM30 with salt stress‐related proteins (GmLEA1 and GmLEC1), and that there were also differences in transcriptional responses between the two haplotypes via RNA‐seq analysis of transgenic hairy roots from *HapT*‐OE and *HapC*‐OE lines under 150 mm NaCl treatment. Moreover, the reason the elite allele *GmPM30‐HapT* has stronger salt tolerance than *HapC* is its stronger interaction within the GmPM30‐lectin (GmLEC1)/GmLEA1 module, which can reduce ion leakage, content of malondialdehyde (MDA), and accumulation of hydrogen peroxide (H_2_O_2_) under salt stress. Further population genetic analyses showed that the low frequencies of the elite haplotypes of *GmLEA1* and *GmLEC1* in improved cultivars indicate their great potential in future breeding, and indicated that elite *GmPM30* alleles have undergone artificial selection to enhance soybean adaptation to high‐latitude regions with high saline‐alkali stress. Field trials revealed that pyramiding the interaction module's elite alleles improves salt tolerance and grain yield of soybean under saline‐alkaline conditions. Finally, we developed an efficient Kompetitive Allele Specific PCR (KASP) marker and utilized this marker for molecular marker‐assisted (MAS) breeding in the F_3_ hybrid population, and identified multiple elite inbred lines carrying the *HapT* allele. This approach indicates that in future breeding programs, it is possible to make full use of MAS to pyramid three elite alleles within the GmLEA1‐GmPM30‐GmLEC1 interaction module, thus accelerating the integration of multiple superior traits.

## 2 Results

### 2.1 Characterization of *GmPM30*

To determine the evolutionary conservation of *GmPM30*, we performed a phylogenetic analysis using proteins with high similarity to GmPM30. The phylogenetic analysis revealed that GmPM30 was closely related to a Group 3 LEA protein Medtr4g011230 (*Medicago truncatula*), a known clade that stabilizes membranes and proteins, and binds ions under dehydration (Figure S1a, Supporting Information). These findings further support *GmPM30*’s conserved role in stress adaptation.

To confirm the potential role of *GmPM30* in abiotic responses, we examined its expression level when soybean seedlings were exposed to different abiotic stresses by reverse transcription quantitative PCR (RT‐qPCR). *GmPM30* transcript levels rose in response to NaCl, H_2_O_2_, and abscisic acid (ABA) treatments. ABA treatment led to a constant increase over time, while NaCl and H_2_O_2_ showed an initial increase followed by a decline (Figure S1b,c,e, Supporting Information). In addition, the sharp rise in *GmPM30* transcript levels observed under NaCl treatment was almost completely suppressed when the seedlings were co‐treated with the ABA biosynthetic inhibitor fluridone (FLU) or the Nicotinamide Adenine Dinucleotide Phosphate (NADPH) oxidase inhibitor diphenyliodonium chloride (DPI) (Figure S1d,f, Supporting Information). This result suggests that the salinity‐induced *GmPM30* expression is likely dependent on ABA and H_2_O_2_. To explore the spatial and temporal expression pattern of *GmPM30*, we performed an RT‐qPCR analysis in different tissues and at different developmental stages, revealing high expression levels in roots at the germination stage (Figure S1g, Supporting Information). *GmPM30* expression levels increased dramatically during the development stage of seeds, which is consistent with the characteristics of LEA proteins being abundant during late embryogenesis. To examine the subcellular localization of GmPM30, we infiltrated *Nicotiana benthamiana* leaves with a GFP‐GmPM30 construct encoding a fusion between GmPM30 and the green fluorescent protein (GFP) and driven by the cauliflower mosaic virus (CaMV) 35S promoter. Fluorescence examination under a confocal microscope demonstrated that *GFP‐GmPM30* localizes to the nucleus and the endoplasmic reticulum (ER), as evidenced by the colocalization of *GFP‐GmPM30* with the nuclear marker P2300 and the ER marker mScarlet‐HDEL (Figure S1h, Supporting Information).

### 2.2 *GmPM30* Promotes Salt Tolerance in Soybean

To assess the role of *GmPM30* in salinity tolerance, we generated transgenic soybean hairy roots overexpressing (*GmPM30‐*OE) or knocked down (*GmPM30*‐RNAi) *GmPM30* in the cultivar Williams 82. We examined the phenotypes of 3‐week‐old *GmPM30‐*OE and *GmPM30‐*RNAi transgenic soybean hairy root plants subjected to three waterings with a 150 mm NaCl treatment over 1 week. When challenged with 150 mm NaCl, *GmPM30‐*OE plants produced larger leaves and more fresh and dry biomass than the EV control, while *GmPM30*‐RNAi lines showed the opposite phenotype (Figure S2a–f, Supporting Information). Importantly, under water treatment only, the EV, *GmPM30‐*OE, and *GmPM30‐*RNAi lines exhibited comparable visible phenotypes and accumulated similar amounts of fresh and dry weight (Figure S2a–f, Supporting Information). Thus, the overexpression of *GmPM30* confers greater salinity tolerance (Figure S2b,c,e,f, Supporting Information). We also examined the activity of superoxide dismutase (SOD) and peroxidase (POD), the photosystem II photochemical potential (*Fv/Fm*), and the concentration of malonaldehyde (MDA), an indicator of lipid peroxidation, in the tissues of *GmPM30‐*OE lines exposed to mock conditions (water only) or salinity (watering with 150 mm NaCl three times over 1 week). When challenged with 150 mm NaCl, the *Fv/Fm*, SOD activity, and POD activity were all higher in *GmPM30‐*OE lines than in the EV control; conversely, MDA in *GmPM30‐*OE decreased by about 25% compared with that in EV plants (Figure S2g–j, Supporting Information). Again, none of these stress indicators were different between EV and *GmPM30*‐OE plants under control conditions.

**Figure 1:**
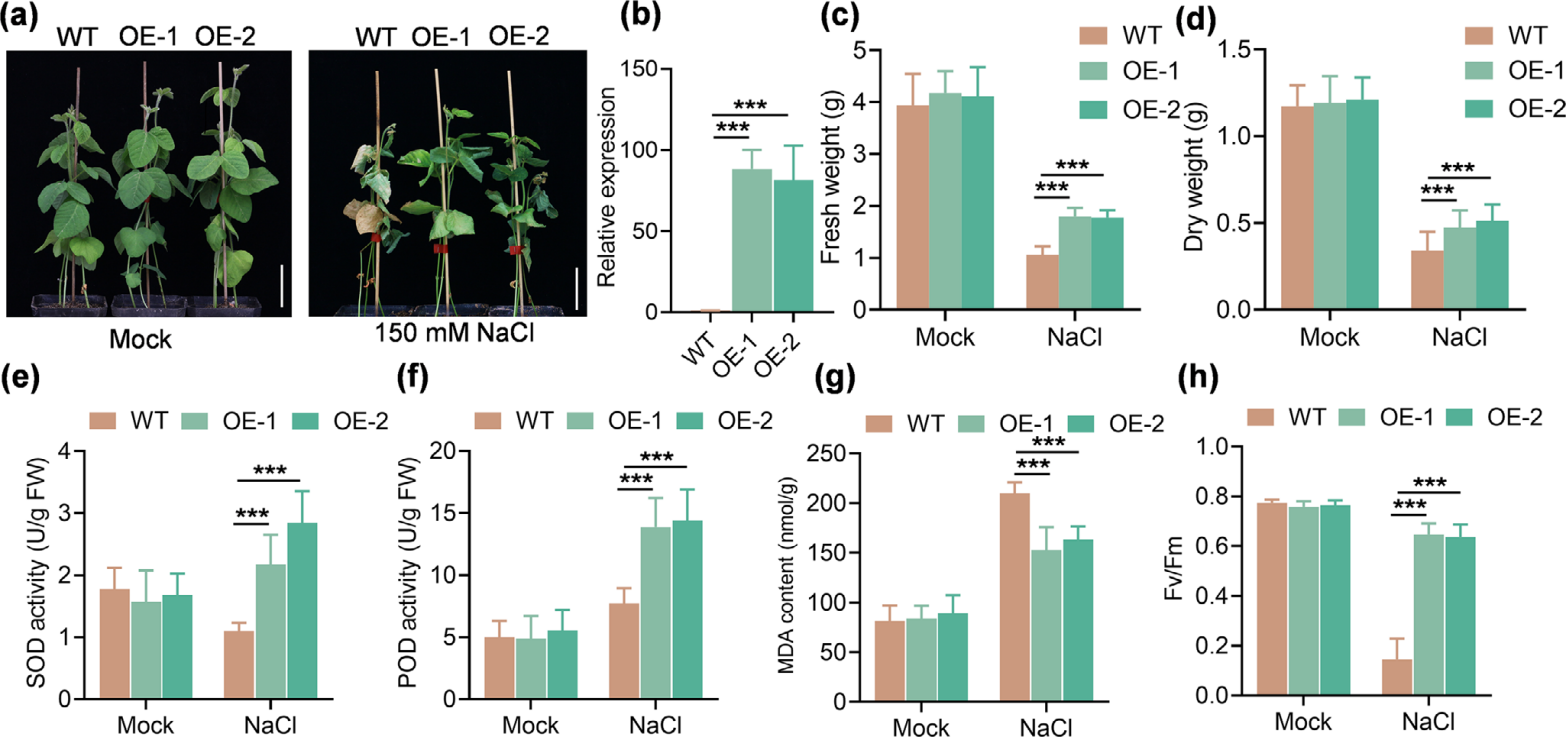
*GmPM30* enhances salt tolerance in soybean transgenic plants. a) Representative images of WT and *GmPM30*‐OE soil‐grown plants at the vegetative stage under mock (H_2_O only) or 150 mm NaCl treatment, taken 14 days after the onset of salinity treatment. Scale bars, 5 cm. b) Expression of *GmPM30* in WT and *GmPM30*‐OE plants. c) Quantification of fresh weight from WT and *GmPM30*‐OE plants. d) Quantification of dry weight from WT and *GmPM30*‐OE plants. e–h) Superoxide dismutase (SOD) activity (e), Peroxidase (POD) activity (f), malondialdehyde (MDA) content (g), and *Fv/Fm* (h) of WT and *GmPM30*‐OE plants under control and 150 mm salt treatment. Data are means ± SD from 10 biological replicates (two‐way ANOVA with ^*^ *p* < 0.05).

Next, to further confirm the biological functions of *GmPM30* in soybean salt tolerance in stable transgenic soybean lines, we examined the phenotypes of 2‐week‐old T_2_‐OE (OE‐1 and OE‐2) lines under 150 mm NaCl treatment for 2 weeks. Under salt stress, the OE lines showed significantly better growth performance, biomass, and physiological indices compared to the wild type, which was consistent with the salt‐tolerant phenotype of transgenic soybean hairy roots (**Figure** **1**; Figure S2, Supporting Information). Together, these results confirm that *GmPM30* significantly enhances salt stress tolerance in soybean and may promote salt tolerance by stabilizing the cell membrane and reducing oxidative damage.

### 2.3 Selection of *GmPM30* Natural Variations Under Adaptation

To understand the evolution of *GmPM30* during domestication, we analyzed the promoter and genomic region of *GmPM30* for evidence of a selective sweep. The Tajima's D value, nucleotide diversity, and *F*_ST_ value revealed a clear selective sweep over *GmPM30*, indicating that *GmPM30* underwent selection during soybean domestication and subsequent genetic improvement (Figure S3a, Supporting Information).

**Figure 2:**
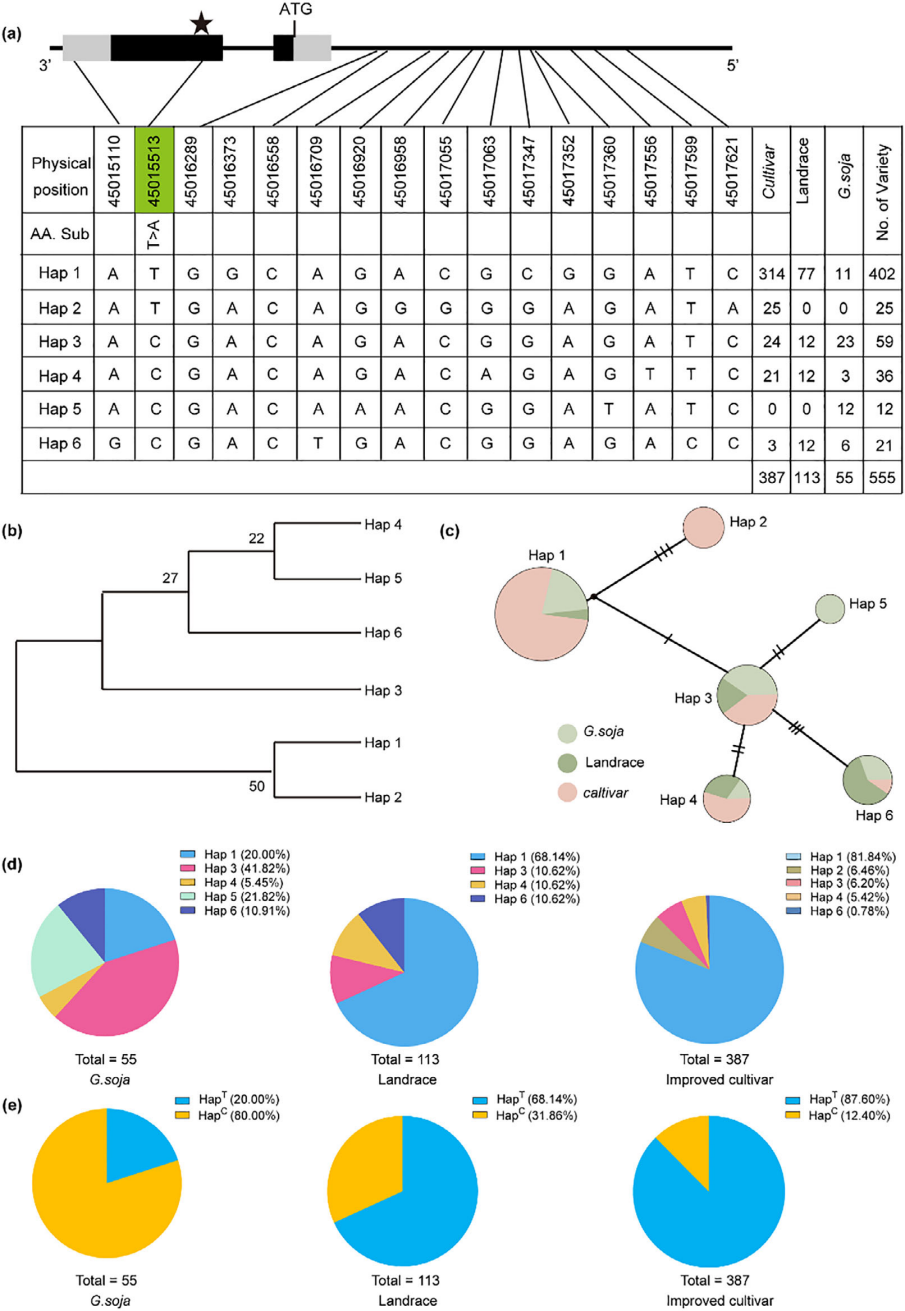
Haplotype analysis of *GmPM30* in Chinese soybean accessions. a) Summary of *GmPM30* haplotypes detected in natural populations. The nucleotide polymorphism in the coding region is indicated by the green box and star. The number of varieties for each haplotype (*Hap1–6*) is shown to the right. b) Evolutionary relationship and origins of the six haplotypes at *GmPM30*. Haplotype origins of *GmPM30* are shown by the median‐joining method. The numbers at each branch represent the percentage support, from 1,000 bootstrap replicates. c) Haplotype network analysis. Circle size is proportional to the number of accessions harboring each haplotype, while circle colors represent the different soybean groups: light green, wild soybean; dark green, landraces; pink, cultivars. d) Distribution of the six main haplotypes at *GmPM30* in wild soybeans, landraces, and improved cultivars. e) Distribution of *GmPM30‐HapT* (corresponding to *Hap1* and *Hap2*) and *GmPM30‐HapC* (corresponding to *Hap3–6*) in wild soybeans, landraces, and improved cultivars.

To investigate the significance of natural variation in *GmPM30* in soybean germplasm resources of China and identify elite alleles, we performed a haplotype analysis of *GmPM30* using 555 soybean accessions. We identified six main haplotypes for *GmPM30*, with *Hap2* only present in cultivated soybeans, while *Hap5* was specific to wild soybeans (**Figure** **2**a). To explore the evolution and spread of the six *GmPM30* haplotypes during soybean domestication and breeding, we reconstructed their corresponding phylogenetic tree and median‐joining haplotype network using the genome sequences of the above 555 accessions. This analysis indicated that *Hap3–6* are closely related, with *Hap3* possibly representing the ancestral haplotype from which all other haplotypes evolved (Figure 2b,c). We also noticed that the frequency of *Hap1* increased from *G. soja* to landraces and from landraces to improved cultivars, suggesting that *Hap1* was strongly and positively selected by humans during soybean domestication and modern breeding efforts (Figure 2d).

Mutations in coding sequences that lead to loss of function often cause strong phenotypic changes;^[^ ^34^, ^35^ ^]^ notably, sequence analysis identified one single‐nucleotide polymorphism (SNP213) in the *GmPM30* coding sequence. *Hap1* and *Hap2* had a T at 211 bp downstream of the ATG of *GmPM30*, while the remaining four haplotypes, *Hap3–6*, had a C at the same position, changing the 72nd amino acid from a threonine into an alanine (T72A), thus defining two major haplotypes designated as *GmPM30‐HapT* (*Hap1*, *Hap2*) and *HapC* (*Hap3–6*). This nonsynonymous SNP reasonably explained the above artificial selection of haplotypes *Hap1* and *Hap2* (*GmPM30‐HapT*) during modern breeding, while *HapC* was gradually eliminated in cultivated soybeans (Figure 2e).

To explore whether natural variation in the *GmPM30* promoter might be responsible for variation in expression levels, we measured *GmPM30* transcript levels in accessions harboring *GmPM30‐HapT* or *GmPM30‐HapC* under control and salinity stress conditions in randomly selected 30 soybean accessions with each haplotype by RT‐qPCR. We observed no significant difference in *GmPM30* expression level between the two haplotypes under control or salinity stress conditions (Figure S4, Supporting Information), suggesting that the sequence polymorphisms in the *GmPM30* promoter do not affect its expression levels.

### 2.4 *HapC* and *HapT* of *GmPM30* Shape Variation in Salt Tolerance and Geographical Adaptation

**Figure 3:**
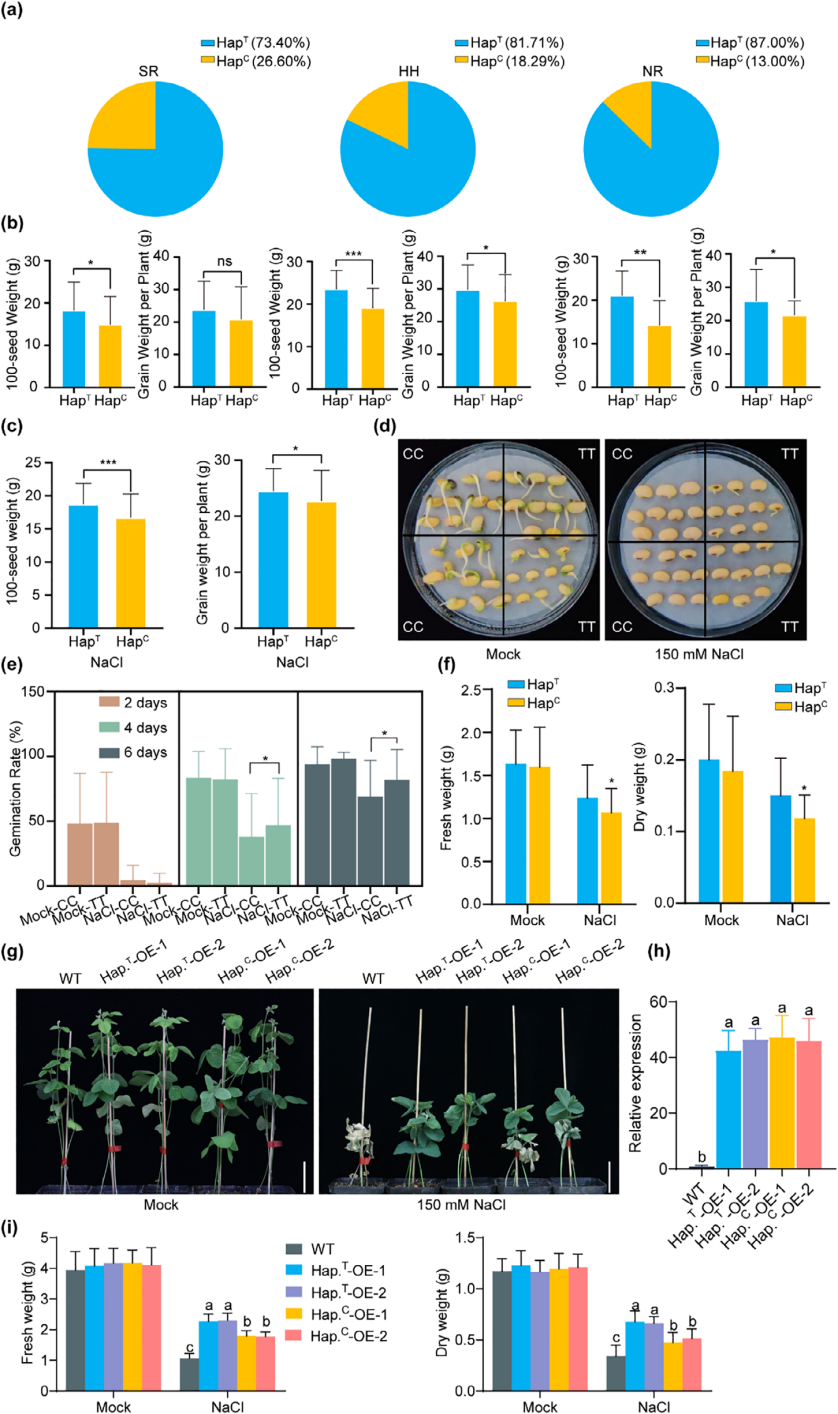
Geographical distribution and functional verification of *HapT* and *HapC* in soybean accessions of China. a) Geographical distribution of the *HapT* and *HapC* alleles. NR, northern region of China; HH, Huang‐Huai region of China; SR; southern region of China. b) Summary of two yield traits, 100‐seed weight and grain weight per plant, for accessions with the *HapT* or *HapC* allele, across low‐and high‐salinity conditions. Each pair of graphs corresponds to the region indicated above it in (a) c) Summary of the two yield traits for plants in saline soils with the *HapT* or *HapC* allele. d) Representative photographs showing the effect of salinity on the germination of seeds from accessions harboring the *HapT* or *HapC* allele. e) Summary of the effect of salinity on the germination rate of accessions in (d). f) Fresh weight and dry weight of accessions with the *HapT* or *HapC* allele under salinity stress. g) Representative photographs of soil‐grown control WT, *HapT*‐OE, and *HapC*‐OE transgenic soybean plants under mock treatment (H_2_O) or NaCl treatment. Scale bars, 5 cm. h) Expression of *GmPM30* in WT, *HapT*‐OE, and *HapC*‐OE plants. i) Fresh weight and dry weight from the transgenic soybean plants shown in (g) with the *HapT* or *HapC* allele under mock or salinity stress conditions.

Previous studies reported that saline‐alkali soils in China are mainly distributed in high‐latitude regions such as the northern (NR), northeast (NE), and Huang–Huai (HR) regions.^[^ ^19^ ^]^ We speculated that natural variation in *GmPM30* may be related to the degree of salinity stress tolerance and latitudinal adaptation across different ecological regions. Indeed, *HapC* of *GmPM30* was more abundant (26.6%) in the southern region (SR), which experiences weaker salinity stress. By contrast, *GmPM30‐HapT* was more enriched in HR (81.7%) and NR (87.0%), which experience more pronounced salinity stress and are located at higher latitudes (**Figure** **3**a).

To better clarify whether accessions with *GmPM30‐HapT* or *HapC* differ from each other under natural conditions at the mature stage and identify elite genotypes that might be deployed in modern breeding, we determined the yield traits and salinity tolerance of about 300 soybean accessions, each carrying one of the two *GmPM30* haplotypes, when grown in the Weifang region of Shandong Province and in the Dongying region of Shandong Province (0.3 g of total soluble salts per 100 g of dry soil). Soybean accessions harboring *GmPM30‐HapT* had higher 100‐seed weight and grain weight per plant than those carrying *HapC* in either ecological region (Figure 3b). We also investigated the yield performance in accessions carrying different haplotypes in saline‐alkali soils, which revealed that the 100‐seed weight and grain weight per plant of *GmPM30‐HapT* accessions under salinity stress in the Dongying region of Shandong Province were each significantly higher than those of accessions with *HapC* (Figure 3c).

To assess the role of the two haplotypes in salinity stress tolerance at the germination and seedling stages, we randomly chose 30 soybean accessions with each haplotype and exposed them to a salinity treatment consisting of watering with 150 mm NaCl. Seed germination rates were equally low in accessions carrying either haplotype when treated with salt stress for 2 or 4 days, while accessions with *GmPM30*‐*HapT* showed a higher seed germination rate than *GmPM30*‐*HapC* accessions at 6 days into salinity stress treatment (Figure 3d,e). We also observed that *GmPM30‐HapT* accessions produced significantly more fresh and dry biomass than *GmPM30‐HapC* accessions at the seedling stage under salinity stress, but not under control conditions (Figure 3f), further supporting the notion that *HapT*‐carrying accessions outperform *HapC*‐carrying accessions for these traits.

To gain functional insight into how *GmPM30‐HapT* and *GmPM30‐HapC* differentially modulate the soybean salt‐stress response, we generated transgenic soybean plant lines overexpressing each haplotype, yielding T_2_ *HapT*‐OE and *HapC*‐OE soybean lines (Figure 3g). Then we examined the relative expression levels of *GmPM30* in *HapT*‐OE and *HapC*‐OE lines to ensure the reliability of the phenotype analysis. *HapT*‐OE and *HapC*‐OE lines with similar expression levels (no significant differences) were selected for the salinity tolerance assay (Figure 3h). Phenotypic analysis revealed no obvious differences under control treatment among the plants with overexpressing *HapT*‐OE and *HapC*‐OE lines; however, plants whose roots overexpressed *GmPM30‐HapT* all displayed higher fresh and dry weight than the *HapC* lines upon treatment with 150 mm NaCl (Figure 3i). Importantly, *HapC*‐OE also plays a positive role in salt resistance; however, its contribution is less than that of *GmPM30‐HapT* based on growth performance, biomass, and physiological indices (Figure 3g,i; Figure S5, Supporting Information). Additionally, we generated 5 transgenic soybean hairy root lines with *GmPM30* knocked down (*GmPM30*‐RNAi) and 4 knocked out (*gmpm30‐ko*) derived from different *HapT*‐containing cultivars. We examined the phenotypes of these *GmPM30‐HapT*‐RNAi and *gmpm30‐ko* transgenic soybean hairy roots, which were subjected to three waterings with 150 mm NaCl over 1 week. The results revealed that 5 *GmPM30‐HapT*‐RNAi and 4 *gmpm30‐ko* transgenic soybean hairy root lines exhibited significantly poorer growth performance and reduced biomass compared with the EV control under 150 mm NaCl treatment (Figure S6a,b, Supporting Information). Together, these results indicated that knocking down and knocking out *GmPM30‐HapT* confer greater salinity sensitivity.

In summary, we speculate that *HapT* of *GmPM30* has high yield potential under salinity stress conditions. Because of its beneficial effect on the adaptation of soybean to higher latitudes and high‐salinity regions, the elite allele *GmPM30‐HapT* might have been artificially selected during modern soybean breeding.

### 2.5 GmPM30 Interacts with GmLEA1 and GmLEC1

**Figure 4:**
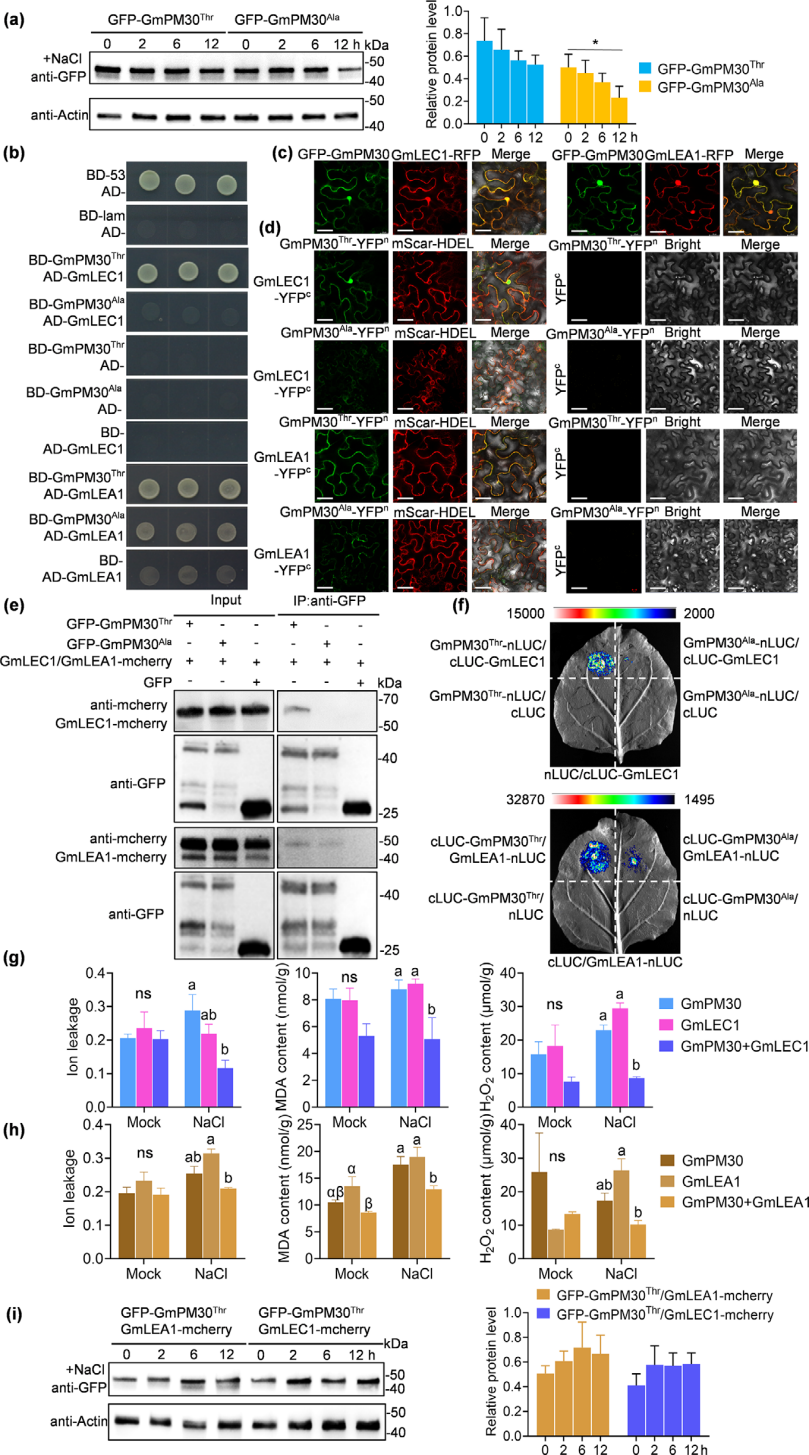
GmPM30 interacts with GmLEA1 and GmLEC1 and synergistically reduces membrane damage. a) The transgenic soybean hairy roots expressing GFP‐GmPM30^Thr^ and GFP‐GmPM30^Ala^ were treated with 150 mm NaCl from 0–12 h. b) Yeast two‐hybrid assay showing the interaction of GmPM30 with GmLEA1 and GmLEC1. Yeast colonies harboring the indicated constructs were grown on synthetic defined medium lacking Leu (L), Trp (T), His (H), and Ade (A). The formation of yeast indicates interaction between the indicated proteins. c) GmPM30 co‐localizes with GmLEA1 and GmLEC1 in *N. benthamiana* cells. Green fluorescence represents the localization of GmPM30, and red fluorescence represents the localization of GmLEA1 and GmLEC1. Scale bars, 40 µm. d) Bimolecular fluorescence complementation (BiFC) assays showing that GmPM30^Thr^ interacts with GmLEA1 and GmLEC1 in *N. benthamiana* leaves were co‐infiltrated with GmPM30 ^Thr^ ‐nYFP and GmLEA1‐cYFP or GmLEC1‐cYFP. Scale bars, 40 µm. e) Co‐immunoprecipitation (Co‐IP) assays, performed in *N. benthamiana* leaves co‐infiltrated with GmLEC1‐mCherry or GmLEA1‐mCherry and GFP‐GmPM30^Thr^, GFP‐GmPM30^Ala^, or GFP. Total proteins extracted from the leaves were immunoprecipitated with anti‐GFP antibody‐conjugated beads, and the precipitates were probed using anti‐mCherry antibody. f) Luciferase complementation imaging (LCI) assays were performed in *N. benthamiana* leaves co‐infiltrated with either cLUC‐GmLEC1 and GmPM30^Thr^‐nLUC/GmPM30^Ala^‐nLUC, or GmLEA1‐nLUC and cLUC‐GmPM30^Thr^/cLUC‐GmPM30^Ala^. g,h) Ion leakage, MDA content, and Hydrogen peroxide (H_2_O_2_) of *N. benthamiana* leaves, which were co‐infiltrated with GmPM30 and GmLEC1 (g) or GmLEA1 (h) under control and salt stress. Data are means ± SD from 3 biological replicates. Lowercase letters and Greek letters indicate significant differences between samples, as determined by 2‐way ANOVA with *p* < 0.05. i) The transgenic soybean hairy roots co‐expressing GFP‐GmPM30^Thr^ and GmLEA1‐mCherry/ GmLEC1‐mCherry were treated with 150 mm NaCl from 0–12 h.

To confirm the biological functions of the T/C polymorphism of *GmPM30* in soybean salt tolerance, we analyzed the protein levels of GmPM30 in different haplotypes under 150 mm NaCl treatment using a hairy root system. The results showed that after 12 h of 150 mm NaCl treatment, the protein abundance of GmPM30‐HapT was significantly higher than that of GmPM30‐HapC (**Figure** **4**a), indicating that the T/C polymorphism alters protein stability.

To understand how *GmPM30* improves plant tolerance to salinity stress in soybean, we carried out a yeast two‐hybrid (Y2H) screen against a cDNA library from the multiple tissues of whole plants and identified 76 unique cDNA clones corresponding to 28 genes (Table S2, Supporting Information). Among the potential candidates, we identified many more clones encoding two proteins, named GmLEA1 and GmLEC1 in this study, than other genes, highlighting their potential importance. Sequence analysis revealed that *GmLEA1* is highly similar to a LEA protein from *G. soja*, while its encoding gene is annotated as having an unknown function in *G. max*. Wild soybeans are generally considered to have a better ability to cope with stress than cultivated soybeans.^[^ ^36^ ^]^ *GmLEC1* contains a legume lectin domain, which participates in protein folding and transportation in the ER, and was predicted to confer salinity tolerance to soybean plants in a previous study.^[^ ^37^, ^38^ ^]^

To validate the potential interaction between these proteins, we performed targeted yeast two‐hybrid assays: GmPM30 indeed interacted with GmLEA1 and GmLEC1 in yeast (Figure 4b). To further determine whether they interacted in vivo, we also conducted bimolecular fluorescence complementation (BiFC) assays, co‐immunoprecipitation (Co‐IP), and luciferase complementation imaging (LCI) assays in *N. benthamiana* leaves co‐infiltrated with the appropriate pairs of constructs. We first performed a colocalization analysis by transiently expressing GFP‐GmPM30 and GmLEA1‐RFP or GmLEC1‐RFP in *N. benthamiana* epidermal cells. This analysis revealed overlapping GFP and RFP signals within cells (Figure 4c), suggesting that GmPM30 might interact with GmLEA1 and GmLEC1. Subsequently, to obtain more direct evidence of the interaction, we performed confocal microscopy analysis. Confocal microscopy examination detected signals from reconstituted yellow fluorescent protein (YFP) when GmPM30‐YFP^N^ (encoding a fusion of GmPM30 to the N‐terminal half of YFP) was co‐expressed with GmLEA1‐YFP^C^ (encoding a fusion of GmLEA1 to the C‐terminal half of YFP) or GmLEC1‐YFP^C^ in *N. benthamiana* leaves (Figure 4d). Our Co‐IP and LCI assays further confirmed the physical association between GmPM30 and GmLEC1/GmLEA1 in vivo (Figure 4e,f). Collectively, these results provide evidence that GmPM30 interacts with GmLEA1 and GmLEC1 in vivo and in vitro.

To investigate whether the C or T polymorphism in the *GmPM30* coding sequence might affect protein function in salinity tolerance by modulating affinity for GmLEA1 and/or GmLEC1. Accordingly, we performed yeast two‐hybrid, BiFC, Co‐IP, and LCI assays to test the interactions of GmPM30 derived from *HapT* or *HapC* with GmLEC1 and GmLEA1. Notably, GmLEC1 and GmLEA1 strongly interacted with the GmPM30‐HapT, but interacted weakly with GmPM30‐HapC (Figure 4b,d,e,f). We further determined the ion leakage, MDA content, and H_2_O_2_ accumulation of the appropriate pairs of interacting constructs under salt stress. When compared with ion leakage, MDA content, and H_2_O_2_ accumulation in *N. benthamiana* leaves infiltrated with the *35S: GFP‐GmPM30* or *35S: GmLEC1‐mCherry* constructs alone, all these indices were decreased when appropriate pairs of *35S: GmPM30* and *35S: GmLEC1* constructs were co‐expressed, because of protein interactions (Figure 4g,h). We obtained similar results for *35S: GmPM30* and *35S: GmLEA1*, indicating that protein interactions synergistically reduced ion leakage, MDA content, and H_2_O_2_ accumulation (Figure 4g,h).

To investigate the effects of the T/C polymorphism in *GmPM30* on protein function, we predicted the structures of different *GmPM30* haplotypes and their interactions with GmLEA1 or GmLEC1 using AlphaFold3. AlphaFold3 predictions indicated that the HapT (GmPM30^Thr^) ‐GmLEA1 and HapT‐GmLEC1 interactions are spatially closer than the HapC (GmPM30^Ala^) ‐GmLEA1 and HapC‐GmLEC1 interactions (Figure, Supporting Information). Quantitative analysis of intermolecular distances showed that in HapT‐GmLEA1 interactions, the Å values were approximately 1/3 of those of HapC‐GmLEA1, and those of HapT‐GmLEC1 were approximately 1/2 of those of HapC‐GmLEC1 (Figure, Supporting Information). Additionally, we observed that the T/C polymorphism altered GmPM30 protein conformation in both HapT‐GmLEA1 and HapC‐GmLEA1 interactions.

To assess how interactions with GmLEA1 or GmLEC1 affect GmPM30‐HapT stability under salt stress, we analyzed GmPM30‐HapT abundance when co‐infiltrated with GmLEA1 or GmLEC1 under salt treatment across different time periods (see new Figure 4i). The results showed that the abundance of GmPM30‐HapT remained stable when co‐infiltrated with GmLEA1 or GmLEC1 under salt treatment across different time periods (see new Figure 4i), indicating that the maintenance of GmPM30‐HapT stability under NaCl treatment depends on its interaction with GmLEA1 or GmLEC1.

Given the interaction between these proteins and GmPM30, we generated transgenic soybean hairy roots overexpressing *GmLEA1 (GmLEA1‐*OE) or *GmLEC1 (GmLEC1‐*OE) to investigate whether *GmLEC1* and *GmLEA1* are involved in salinity stress responses. Phenotypic analysis revealed that *GmLEA1‐*OE and *GmLEC1‐*OE plants are significantly more tolerant to salinity stress than the EV control plants under NaCl treatment, confirming that these two genes are indeed involved in salinity tolerance in soybean (Figure S5g, Supporting Information). These results also suggest that *GmPM30* may play a synergistic role with *GmLEC1* and *GmLEA1* in enhancing salinity tolerance.

### 2.6 *GmLEA1* and *GmLEC1* are Also Under Selection During Domestication of Soybean

To determine whether *GmLEA1* and *GmLEC1* were also under selection, we calculated F_ST_, nucleotide diversity, and Tajima's D values between wild soybeans and cultivars. We obtained similar results for the F_ST_, nucleotide diversity, and Tajima's D values compared with *GmPM30* (Figure S3b,c, Supporting Information), suggesting that *GmLEA1* and *GmLEC1* have been retained in cultivars through positive selection during domestication, which may promote the adaptation of cultivars to salt stress.

To investigate the origin and evolution of *GmLEA1* and *GmLEC1* during soybean domestication, we performed haplotype analysis of *GmLEA1* and *GmLEC1*. The results showed that there were five major haplotypes of *GmLEA1* in 538 soybean germplasms following sample quality control and screening (Figure S7a, Supporting Information). The frequency of *Hap1* and *Hap3* increased initially and then decreased from wild soybeans to landraces and cultivars, while the frequency of *Hap2* decreased first and then increased; however, it rose from wild soybeans to cultivars, indicating artificial selection of *Hap1*‐*3* during domestication and modern breeding (Figure S7b, Supporting Information). To explore the evolution and spread of the five *GmLEA1* haplotypes during soybean domestication and breeding, we reconstructed their median‐joining haplotype network using the genome sequences of the above 538 accessions. Haplotype network analysis indicated close relationships between *Hap1*–*2* and *Hap3–5*. *Hap4*, a mixed group of wild, landrace, and cultivated soybeans, likely represents the ancestral haplotype from which others evolved, suggesting bidirectional gene flow during domestication (Figure S7c, Supporting Information). Haplotype‐phenotype association analysis showed that, as the elite haplotype of *GmLEA1*, *Hap3* exhibited higher grain yield (grain weight per plant and 100‐seed weight) under salinity stress than other haplotypes at the mature stage (Figure S7d,e, Supporting Information). We also noticed the lower frequency of *Hap3* in improved cultivars, which highlights its high breeding value in modern breeding.

Furthermore, after sample quality control and screening, haplotype analysis revealed that *GmLEC1* had four haplotypes in wild soybeans and three in both landraces and cultivars across 517 soybean accessions (Figure S7f, Supporting Information). Except for *Hap4*, which is specific to wild soybeans, the frequency of other haplotypes increased from wild soybeans to improved cultivars, indicating strong and positive selection of *Hap1*‐*3* during domestication and breeding (Figure S7g, Supporting Information). Haplotype network results showed that *Hap1*‐*3* of *GmLEC1* directly originated from wild soybeans, differing from the differentiation form of *GmLEA1* (Figure S7h, Supporting Information). Although the frequency of elite *Hap3*, which enhanced grain yield (grain weight per plant and 100‐seed weight) under salinity stress, increased across wild soybeans, landraces, and improved cultivars, its relatively low frequency (21.82%) in improved cultivars also suggests great breeding potential (Figure S7i,j, Supporting Information).

### 2.7 Transcriptome Analysis of Different Haplotypes of GmPM30 Overexpressing Transgenic Soybean Hairy Roots

To further understand the molecular mechanisms by which different haplotypes of *GmPM30* regulate salt‐stress tolerance, we performed a transcriptome sequencing analysis (RNA‐seq) of transgenic *GmPM30‐HapT*, *GmPM30‐HapC*, and EV hairy roots.

**Figure 5:**
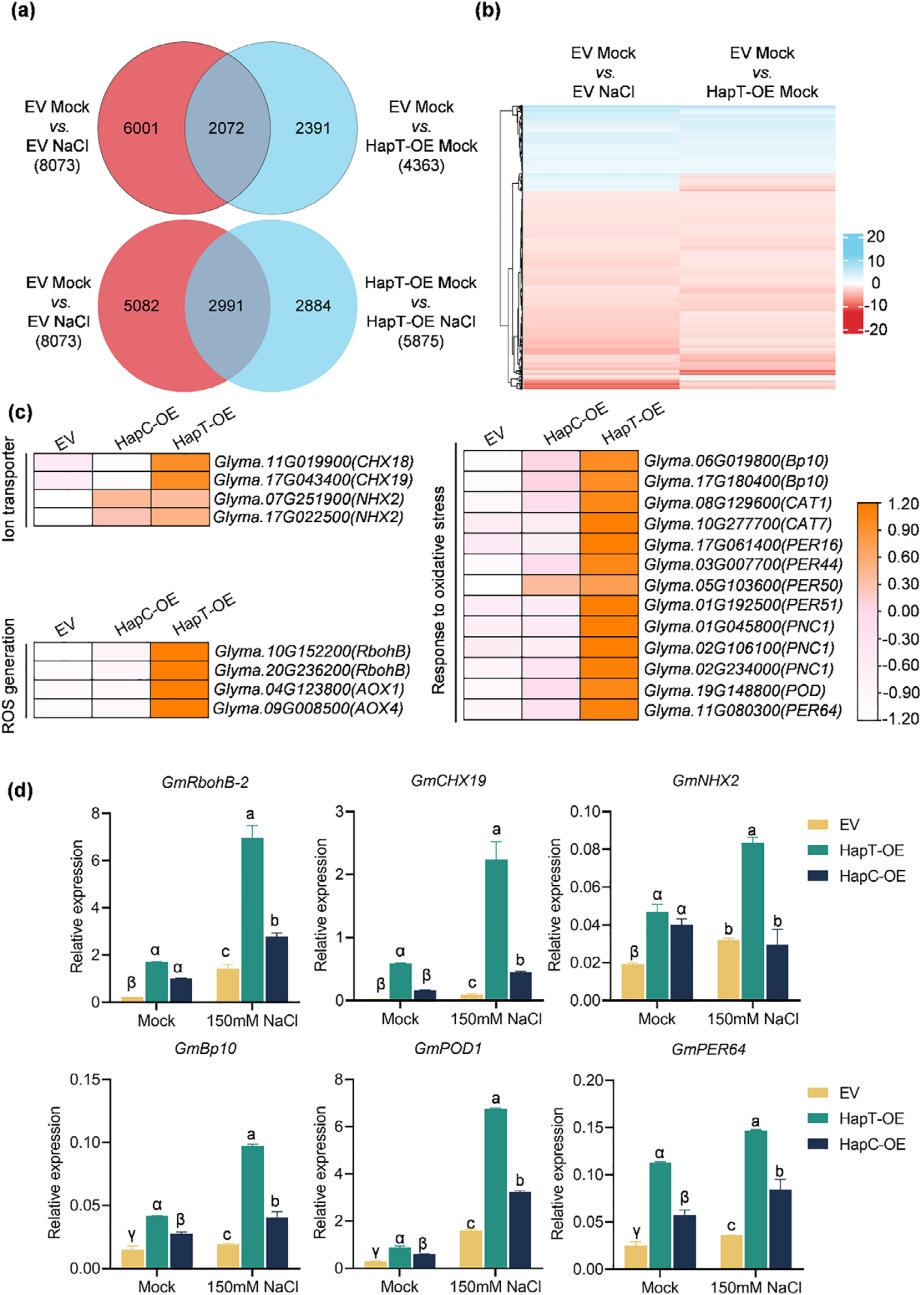
Transcriptome analysis of *HapT*‐OE, *HapC*‐OE, and EV transgenic soybean hairy roots. a) Venn diagrams showing the extent of overlap for DEGs between *HapT*‐OE and EV transgenic soybean hairy roots under mock and salinity stress conditions. b) Hierarchical cluster analysis of the overlapping genes from (a) differentially expressed in mock‐treated EV versus NaCl‐treated EV and mock‐treated EV versus mock‐treated *HapT*‐OE. The numerical values in the gradient bar represent log2 (fold change) relative to the control sample. c) Heatmap representation of expression levels for ROS generation, ion transport‐related genes, and responding to oxidative stress genes in EV versus *HapC*‐OE versus *HapT*‐OE. Values are log2 (fold change). d) Relative transcript levels of *GmRbohB‐2*, *GmCHX19*, *GmNHX2*, *GmBp10*, *GmPOD1*, and *GmPER64* in the hairy roots of EV, *HapC*‐OE, and *HapT*‐OE exposed to no NaCl (0 h) and 150 mm NaCl for 2h. Error bars denote SD (*n* = 3 from 3 biological experiments). Lowercase letters and Greek letters indicate significant differences between samples, as determined by 2‐way ANOVA with *p* < 0.05.

To ensure analysis results reliability, we validated overexpression levels across haplotypes using three primer pairs targeting *GmPM30*, *GFP*, and *Bar* genes (Figure S8a, Supporting Information). Two biological replicates per group (EV, *HapT*, *HapC*) with no significant expression differences were selected for RNA‐seq. These samples were subjected to 0 and 150 mm NaCl treatment for 0 h and 2 h, with three biological replicates per time point. A total of 8073 differentially expressed genes (DEGs; 2394 up‐regulated and 5679 down‐regulated) were identified in the comparison between mock‐and NaCl‐treated EV hairy roots. However, NaCl treatment did not cause a remarkable transcriptomic change in *GmPM30‐HapT* hairy roots relative to EV, with only 5875 genes differentially regulated (**Figure** **5**a); we also identified 7894 DEGs in *GmPM30‐HapC* hairy roots (Figure S8c, Supporting Information). These results suggested that *HapT*‐OE roots are less sensitive to NaCl treatment than *HapC*‐OE and EV roots in terms of transcriptomic changes.

To better understand the relationship of DEGs caused by *GmPM30* different haplotypes overexpression and by NaCl treatment, the expression pattern of the 2072 and 678 DEGs detected common in these comparisons was analyzed using linear regression and hierarchical clustering methods (Figure 5a; Figure S8c, Supporting Information). The expression patterns of some of the DEGs were similar (Figure 5b; Figure S8d, Supporting Information), indicating that some of the transcriptional changes caused by salt stress are mediated by *GmPM30*. A Kyoto Encyclopedia of Genes and Genomes (KEGG) pathway enrichment analysis showed that the DEGs in the mock‐treated *HapT*‐OE and EV hairy roots were enriched in the top six pathways: “Biosynthesis of secondary metabolites,” “Metabolic pathways,” “Phenylpropanoid biosynthesis,” “Cysteine and methionine metabolism,” “Photosynthesis,” and “Flavonoid biosynthesis” (Figure S9a, Supporting Information). In the NaCl‐treated *HapT*‐OE and EV roots, KEGG pathway enrichment analysis showed that DEGs were further enriched in the category “Phenylpropanoid biosynthesis” (Figure S9b, Supporting Information), while in NaCl‐treated *HapC*‐OE and EV roots, DEGs were not further enriched (Figure S9d, Supporting Information). To investigate which biological pathways are involved in the NaCl *HapT*‐OE versus NaCl EV cluster, we performed Gene Ontology (GO) analysis. The cluster was enriched in biological processes related to stress responses, such as “Heme binding,” “Tetrapyrrole binding,” and “Hydrogen peroxide catabolic process” (Figure S10b, Supporting Information). In contrast, GO analysis of NaCl‐treated *HapC*‐OE and EV roots revealed that DEGs were enriched for distinct biological processes, such as the “Transcription factor activity,” “Endopeptidase inhibitor activity,” “Peptidase inhibitor activity,” and “Cell wall” (Figure S10d, Supporting Information), indicating that *GmPM30* is involved in the salt stress response and modulates expression of oxidative stress‐related genes (e.g., *GmRbohB‐2*, *GmPOD1*), whereas *HapT*‐OE extensively and robustly activates stress response pathways.

Further heat maps showed that genes related to ROS generation, ion transport, and responding to oxidative stress were significantly up‐regulated in *HapT*‐OE hairy roots compared to EV under the mock treatment (Figure 5c). We speculated that changes in the expression of these genes might contribute to the salt tolerance of the *HapT*‐OE. Accordingly, we selected the *GmRbohB‐2*, *GmCHX19*, *GmNHX2*, *GmBp10*, *GmPOD1*, and *GmPER64* for further expression levels analysis in the EV, *HapT*‐OE, and *HapC*‐OE hairy roots. This analysis showed that these six selected genes were all up‐regulated in *HapT* and *HapC* compared to EV hairy roots (Figure 5d). Additionally, these genes were also induced by NaCl treatment, and *HapT*‐OE had a higher expression level, further explaining why *HapC*‐OE plants are more vulnerable to salt stress.

### 2.8 Pyramiding of Elite Haplotypes of *GmPM30*, *GmLEA1*, and *GmLEC1* Improves Grain Yield Under Salt Stress

**Figure 6:**
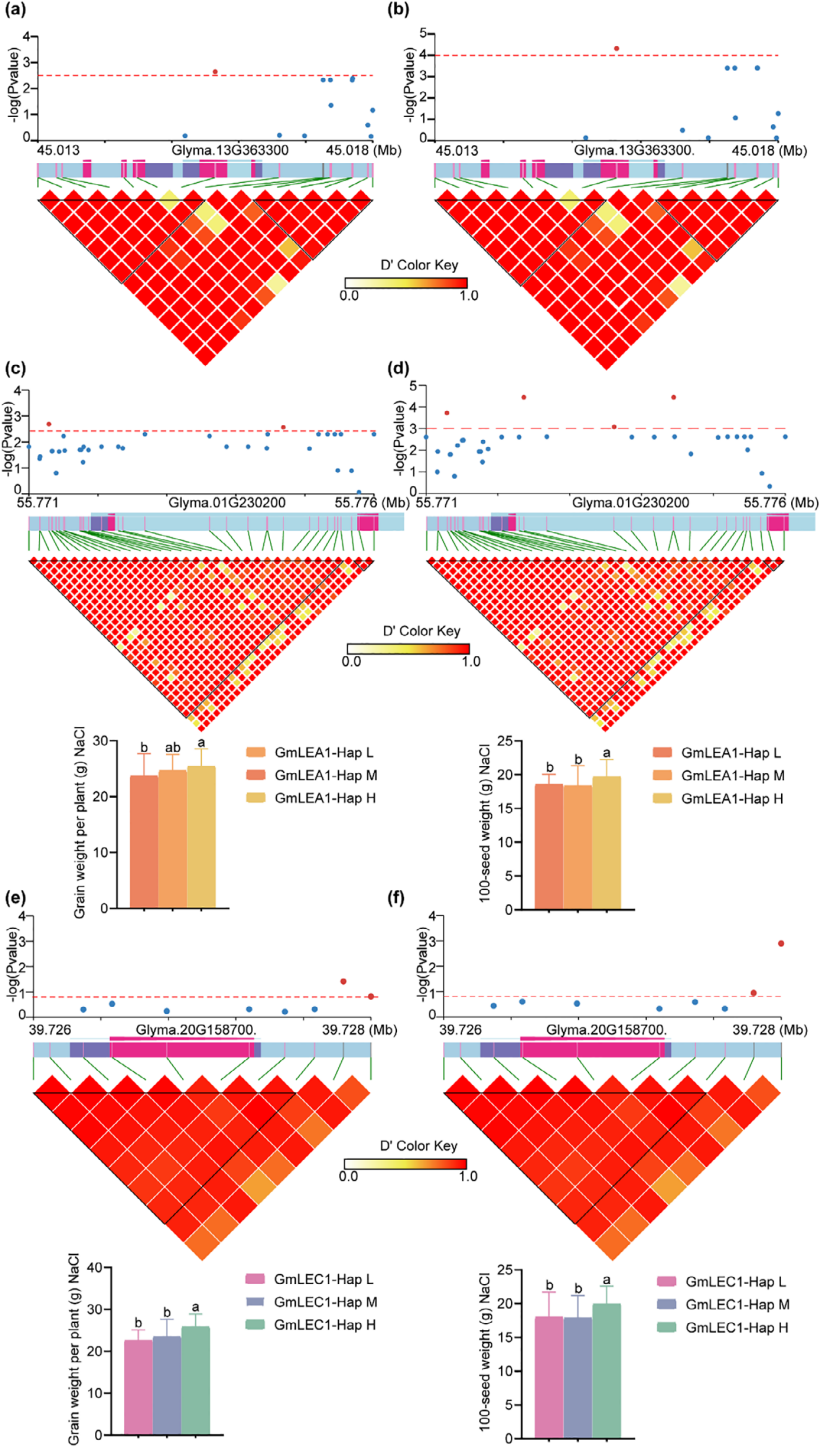
Significant SNPs in the *GmPM30*, *GmLEA1*, and *GmLEC1* confer different grain yield under salt stress. a,c,e) Candidate gene‐based association analysis of *GmPM30* (a), *GmLEA1* (c), and *GmLEC1* (e) using grain weight per plant of cultivars under salt stress. b,d,f) Candidate gene‐based association analysis of *GmPM30* (b), *GmLEA1* (d), and *GmLEC1* (f) using 100‐seed weight of cultivars under salt stress. The histogram represents the genotyping results of significant SNPs. Lowercase letters indicate significant differences between samples, as determined by one‐way ANOVA with ^*^ *p* < 0.05.

To clarify the functional significance and association of the variations among *GmPM30*, *GmLEA1*, and *GmLEC1*, we conducted a candidate gene‐based association analysis using 300 cultivar accessions and found that several SNPs were strongly associated with grain weight per plant and 100‐seed weight under salt stress (**Figure** **6**). To test whether significant SNPs at different positions in the three genes are associated with soybean grain yield under salt stress, we classified *GmPM30* into two haplotypes with high (*Hap‐H*) and low *(Hap‐L)* grain yields and *GmLEA1*/*GmLEC1* into three major haplotypes with high, medium *(Hap‐M)*, and low grain yields according to these highly significant SNPs (Figures 3c,6). We also found that the haplotype allele *Hap‐H* of the three genes significantly contributed to grain yield under salt stress, and accessions harboring *GmPM30‐H*, *GmLEA1‐H*, and *GmLEC1‐H* matched the elite haplotype results from previous analysis. These findings indicate the importance of these associated variations for grain yield.

**Figure 7:**
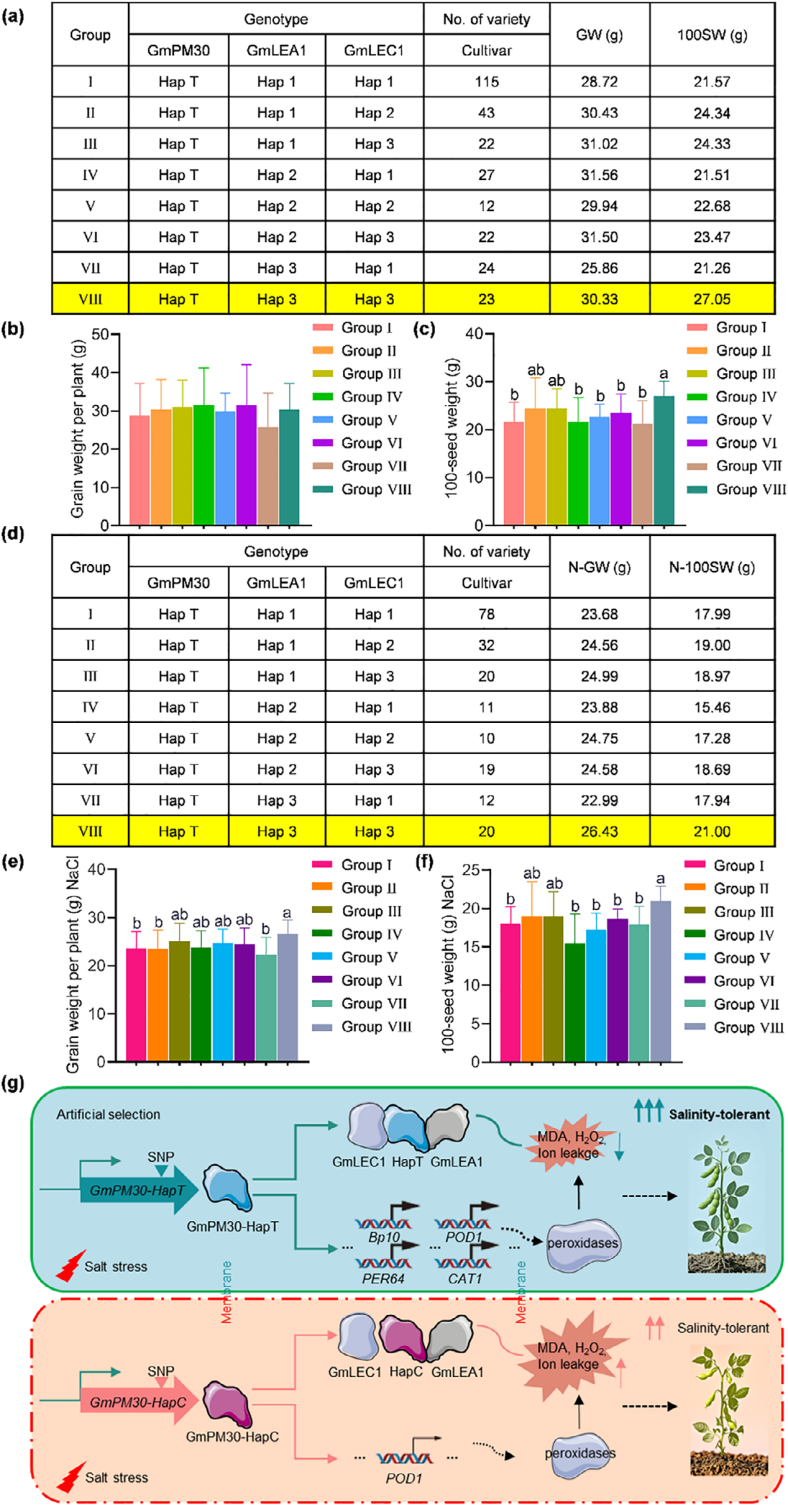
Combined haplotype analysis of *GmPM30*, *GmLEA1*, and *GmLEC1* and its breeding potential. a) Combined haplotypes of *GmPM30*, *GmLEA1* and *GmLEC1* under mock conditions. b,c) Grain weight per plant (b) and 100‐seed weight (c) of haplotypes of different combinations in the cultivars under mock conditions. d) Combined haplotypes of *GmPM30*, *GmLEA1* and *GmLEC1* under salinity conditions. e,f) Grain weight per plant (e) and 100‐seed weight (f) of haplotypes of different combinations in the cultivars under salinity conditions. Data represent means ± SD. Lowercase letters indicate significant differences between samples, as determined by one‐way ANOVA with *p* < 0.05. g) Artificial selection of *GmPM30‐HapT* and its molecular mechanism improves soybean salt tolerance.

Since these three genes, located on different chromosomes, collaboratively regulate soybean grain yield under salt stress, we performed a combined haplotype analysis using their major haplotypes and obtained eight haplotype combinations, designated as groups I–VIII (**Figure** **7**a,d). Different haplotype combinations exhibit distinct phenotypes. Group VIII represents the aggregation of three elite alleles, namely *GmPM30*‐*HapT (H)*‐*GmLEA1*‐*Hap3 (H)*‐*GmLEC1*‐*Hap3 (H)*. Agronomic trait analysis showed that, under normal conditions, the average grain weight per plant for all groups ranges from 25.86 to 31.56 g, and the average 100‐seed weight is between 21.26 and 27.05 g. Among them, the grain weight per plant of Group VIII did not differ significantly compared with other groups, but Group VIII has the third‐highest grain weight per plant and the highest 100‐seed weight, which is significantly higher than that of multiple groups. Under salt stress, the average grain weight per plant for all groups is from 22.99 to 26.43 g, and the average 100‐seed weight ranges from 15.46 to 21.00 g. Although the yield is lower compared to that under normal conditions, Group VIII has the highest grain weight per plant and 100‐seed weight under salt stress, which are significantly higher than those of most other groups (Figure 7b,c,e,f). Moreover, we found that the germplasm of Group VIII included the soybean cultivar Zhonghuang 13 (ZH13), the most widely planted soybean cultivar in China with a rich genetic background and elite agronomic traits. Therefore, pyramiding *GmPM30‐HapT*, *GmLEA1‐Hap3*, and *GmLEC1‐Hap3* would provide abundant genetic resources and a foundational reference for breeding soybeans with high grain yield under salt stress.

To assess the utilization of *GmPM30*, *GmLEA1*, and *GmLEC1* in breeding, we investigated the allele frequencies of *GmPM30*, *GmLEA1*, and *GmLEC1*. *GmPM30‐T* has a high allele frequency in landraces and improved cultivars; thus, *GmPM30‐T* may have been used to improve grain weight and salt tolerance in soybeans during early breeding. The elite allele frequency of *GmLEC1* increased from landraces to improved cultivars, indicating more effective utilization. Moreover, the combined frequency of *GmPM30*/*GmLEA1*/*GmLEC1* elite alleles increased in the improved cultivars, suggesting this haplotype combination's advantage in breeding for high grain yield and salt tolerance.

### 2.9 Introgression of the *GmPM30‐HapT* Improves Yield Performance of Hybrid Lines Under Saline Soil

**Figure 8:**
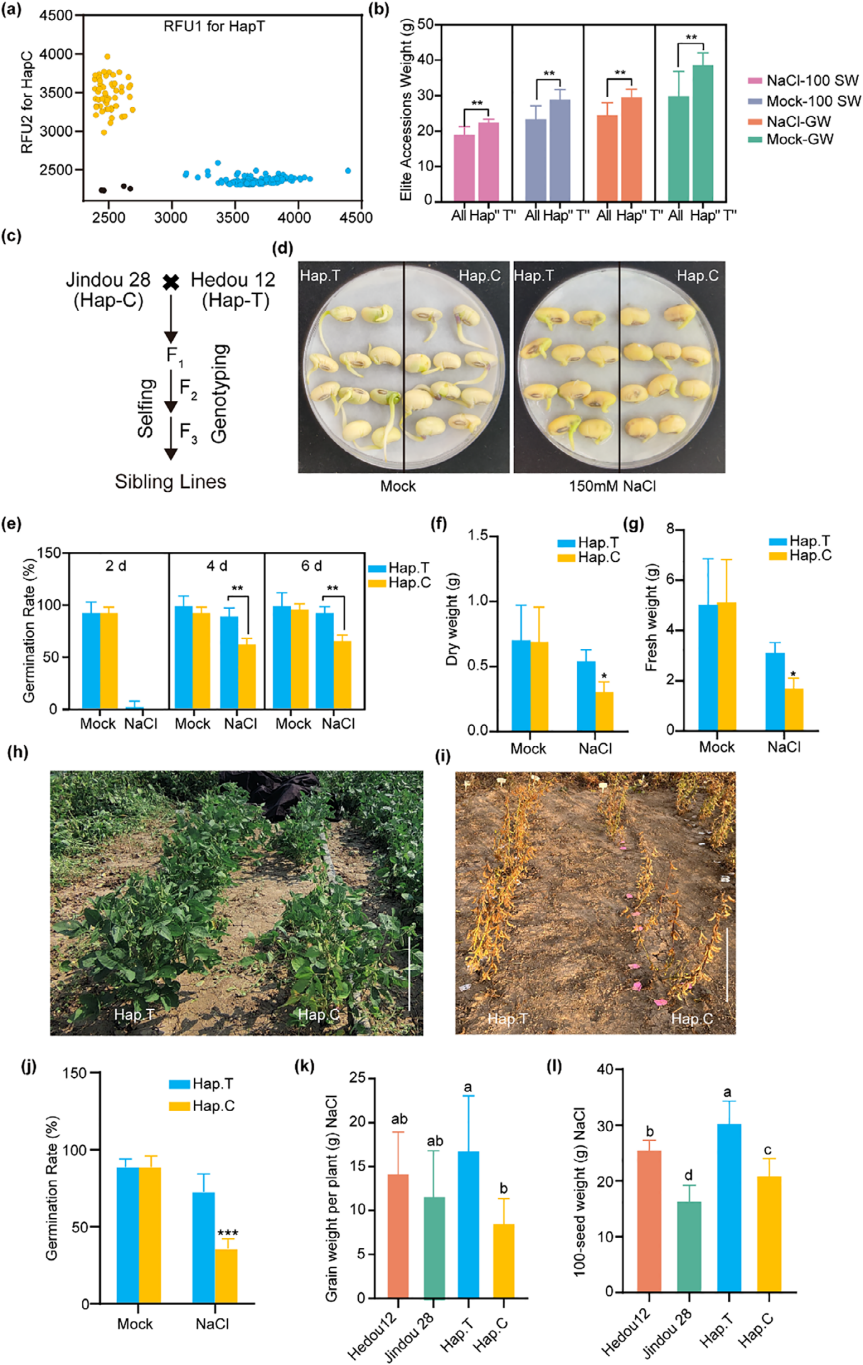
*GmPM30‐HapT* represents an elite allele in modern breeding, which enhances salinity tolerance and yield traits on saline farmlands. a) Genotyping results of the functional KASP marker for *HapC* and *HapT* across soybean accessions. Relative Fluorescence Unit (RFU) is a unit of measurement used in analysis that employs fluorescence detection. b) Yield traits under control and salinity stress conditions for six elite accessions selected based on the KASP functional marker. c) The procedure for introducing sibling lines of different haplotype. d) Representative photographs showing the effect of salinity on the germination of seeds from sibling lines harboring the *HapT* or *HapC* allele. e) The effect of salinity on the germination rate of sibling lines in (d). f) Dry weight of sibling lines with the *HapT* or *HapC* allele under salinity stress. g) Fresh weight of sibling lines with the *HapT* or *HapC* allele under salinity stress. Photos of *GmPM30‐HapT* and *GmPM30‐HapC* lines at the seedling stage h) and he mature stage i) in saline fields in Dongying, China. Scale bars, 40 cm. j) The emergence rate of sibling lines in (h). The grain weight per plant k) and 100‐seed weight l) of *HapT*, *HapC* lines, Jindou 28, and Hedou 12 in saline fields.

To utilize the breeding potential of the *GmPM30‐HapT* allele in grain yield traits under salt stress, we intended to introduce the *GmPM30‐HapT* allele into accessions carrying the *GmPM30‐HapC* allele through hybridization. To rapidly distinguish hybrid lines with different alleles in breeding, we developed a Kompetitive Allele Specific PCR (KASP) related to High Salinity Tolerance and Yield Traits marker named *Kasp‐HSY* to genotype *GmPM30* for the T/C SNP through marker‐assisted selection (MAS). This marker effectively and consistently (100%) distinguished between *HapT* and *HapC* in a panel of 168 randomly selected accessions from the larger panel of 555 accessions used earlier (Table S3, Supporting Information; **Figure** **8**a). Furthermore, we examined the top 30% soybean cultivars harboring *GmPM30‐HapT* based on grain yield, and selected six elite accessions with high yield and strong salinity tolerance (Figure 8b; Table S4, Supporting Information and Figure S11, Supporting Information). Then, the backbone soybean cultivar Hedou 12, exhibiting the best comprehensive performance (top 7%), was utilized to establish the population (Figure 8c).

We used the KASP marker to select sibling lines with different haplotypes in the F_3_ hybrid population (Jindou 28×Hedou 12), and performed salinity tolerance assays in the saline field and greenhouse, respectively (Figure 8c). The results showed that under 150 mm NaCl treatment, hybrid lines harboring *GmPM30‐HapT* showed higher germination rates at 4 to 6 days (Figure 8d,e) and greater seedling biomass (Figure 8f,g) than *GmPM30‐HapC* lines, indicating their superior salt tolerance. We also performed field trials on saline farmlands in Dongying, China (0.3 g of total soluble salts per 100 g of dry soil). At the seedling stage, the survival rate and growth performance of *GmPM30‐HapT* lines were superior to those of the *GmPM30‐HapC* lines (Figure 8h,j). At the mature stage, *GmPM30‐HapT* lines produced superior performance, as measured in both 100‐seed weight and grain weight per plant, compared to *GmPM30‐HapC* lines (Figure 8k,l), suggesting that introgression of the *GmPM30‐HapT* into F_3_ hybrid lines improves soybean yield performance on saline soil. Our findings suggest that *GmPM30* is indeed a valuable target for salinity‐tolerant and high‐yielding soybean breeding.

## 3 Discussion

### 3.1 *GmPM30* Enhances Soybean Salt Tolerance via Natural Variation in Populations

Salinity stress inhibits the normal growth and development of plants, making it a major environmental factor affecting the yield and quality of soybean.^[^ ^30^ ^]^ Identification of soybean salinity tolerance genes and their natural elite allelic variants, as well as their application in molecular design breeding, is an effective way to breed high‐yielding and salinity‐tolerant soybean accessions.^[^ ^12^, ^39^ ^]^ However, although LEA proteins were first characterized 40 years ago in plants,^[^ ^22^ ^]^ studies on the underlying molecular mechanisms through which SNPs of LEA proteins influence their functions in soybean natural populations remain unclear.^[^ ^29^ ^.^ ^30^, ^40^, ^41^ ^]^ Recently, many studies have shown that natural variation in the promoter region is highly associated with transcriptional activity and binding activity of upstream transcription factors.^[^ ^34^, ^42^, ^43^, ^44^, ^45^, ^46^, ^47^ ^]^ However, studies on natural variations in gene coding regions that cause functional protein changes without affecting expression levels remain limited. For example, SNPs introducing premature stop codons in the *E2* coding region result in the formation of two truncated proteins in soybean, e2‐1 (527 amino acids) and e2‐2 (52 amino acids); soybean accessions producing e2‐1 or e2‐2 performed better in terms of salinity tolerance than accessions carrying a functional *E2* allele,^[^ ^19^ ^]^ indicating that the use of loss‐of‐function alleles can create elite plant materials. In maize (*Zea mays*), two nonsynonymous SNPs in the *ZmSRO1d‐S* haplotype *(Similar to RCD1 One 1d Sensitive)* can affect protein localization, thereby influencing protein interactions and promoting plant drought tolerance.^[^ ^48^ ^]^ Here, we demonstrated that *GmPM30* promotes salinity tolerance in soybean and have identified a new elite haplotype, *GmPM30‐HapT*, which causes a Thr‐to‐Ala change in *GmPM30* and leads to higher yield‐related traits and salinity tolerance than *HapC*, providing new insights into how single‐nucleotide variation affects protein function and plant traits (Figures 2, 3).

### 3.2 Haplotype‐Specific Interaction within the GmLEA1‐GmPM30‐GmLEC1 Module Synergistically Enhances Soybean Salt Tolerance

Previous studies have shown that almost all groups of LEAs oligomerize under stress conditions, but this phenomenon has yet to be reported for soybean LEAs.^[^ ^49^ ^]^ The molecular chaperone properties of LEA proteins allow them to interact with proteins to stabilize their structures and functions, maintain cellular osmotic pressure, and preserve membrane integrity. Here, we identified a LEA protein GmLEA1 through yeast two‐hybrid (Y2H) library screening and confirmed that GmPM30 interacts with GmLEA1 protein in vivo and in vitro by Y2H, BiFC, Co‐IP, and LCI (Figure 4b,d,e,f), suggesting that GmPM30 may exert its function via the formation of heterogenic oligomers. Lectin receptor kinases (LecRKs) participate in salinity tolerance and are involved in ABA responses and reactive oxygen species (ROS) scavenging, but a clear regulatory mechanism is lacking.^[^ ^37^, ^50^, ^51^, ^52^, ^53^ ^]^ Lectins bind to the extracellular domain of lectin receptor‐like kinases (LecRLKs) and receptor‐like proteins (LecRLPs). Moreover, within cells, lectins also often participate in protein folding, transport, and stress responses. We also identified a lectin protein, GmLEC1, through yeast two‐hybrid (Y2H) library screening and confirmed its interaction with GmPM30 in vivo and in vitro by Y2H, BiFC, Co‐IP, and LCI (Figure 4b,d,e,f), indicating that *GmPM30* and *GmLEC1* may form a module that promotes salinity tolerance in soybean. Notably, two haplotypes resulting from a nonsynonymous SNP in the coding region of *GmPM30* can affect protein interaction strengths with GmLEC1 and GmLEA1 (Figure 4b,d,e,f), with *GmPM30‐HapT* showing stronger interactions. To confirm the role of GmPM30 interactions with GmLEA1 or GmLEC1 in soybean salt tolerance, we performed single‐gene infiltration assays using *35S: GmPM30*, *35S: GmLEA1*, or *35S: GmLEC1* and co‐expression assays with *35S: GmPM30* paired with *35S: GmLEA1* or *35S: GmLEC1* in *N. benthamiana* leaves. The results showed that, compared with single‐gene expression, the transgenic leaves co‐transfected with *GmPM30*+*GmLEA1* or *GmPM30*+*GmLEC1* synergistically reduced the ion leakage, MDA content, and H_2_O_2_ accumulation (Figure 4g,h). This indicates that the degree of cell membrane lipid oxidation and cell damage in the co‐expressed transgenic leaves was lower than that in the leaves with single‐gene expression. These results suggest that the co‐expressed genes play a synergistic protective role during the salt resistance process of plants. The higher salt tolerance of *GmPM30*‐*HapT* compared with *GmPM30*‐*HapC* may be due to stronger interactions within the GmPM30‐GmLEC1/GmLEA1 module. Therefore, two haplotypes resulting from a nonsynonymous SNP in the coding region of *GmPM30* enhance salt tolerance through different interaction strengths within the GmLEA1‐GmPM30‐GmLEC1 interaction module (Figure 4).

### 3.3 Pyramiding Elite Haplotypes of *GmPM30‐GmLEA1‐GmLEC1* Confers Additive Salt Tolerance and Yield Gains in Soybean

Evolutionary and population genetics analyses suggest that *GmPM30*, *GmLEA1*, and *GmLEC1* were located in the domestication‐selected regions, indicating their common roles during soybean domestication and stress adaptation (Figure S3, Supporting Information). Interestingly, the selection of *GmPM30* was also related to geographical differentiation, suggesting that artificial selection of elite *GmPM30‐HapT* might have contributed to soybean adaptation to high latitudes and saline‐alkaline soils and thereby enhanced grain yield (Figure 3). This evolutionary characteristic highlights their co‐selection for enhanced salt tolerance, providing molecular evidence for the adaptive evolution of the protein‐protein interaction network in cultivated soybeans. More importantly, origin and breeding potential analysis show that the favorable allele *GmPM30‐HapT* had been selected during modern soybean breeding. In contrast, only 15.63% and 21.82% of the improved cultivars examined here carried elite alleles of *GmLEA1* and *GmLEC1* (Figure S7b,g, Supporting Information), suggesting that the favorable alleles of *GmLEA1* and *GmLEC1* are rare variants with potential to be utilized in future soybean breeding. Since traits of interest are often multi‐gene‐regulated, the pyramiding of haplotypes enables rapid and precise identification of superior materials. However, research on the pyramiding of elite haplotypes of salt‐tolerant genes is limited. Our saline‐alkaline field trials also revealed that pyramiding the *GmLEA‐GmPM30‐GmLEC1* module conferred an additive effect under saline‐alkaline conditions: lines combining elite haplotypes of *GmPM30*, *GmLEA1*, and *GmLEC1* haplotypes obtained higher grain yield compared to other lines (Figure 7a–e). Moreover, these elite lines include the soybean backbone cultivar ZH13 (Table S4, Supporting Information), which is the first soybean cultivar in China with an annual promotion area exceeding 10 million mu and a cumulative promotion area exceeding 100 million mu, remaining the cultivar with the broadest adaptation range to date. ZH13 has high and stable yields and has set a yield record of 312.4 kg mu^−1^ in the HH region of China. Therefore, pyramiding *GmPM30‐HapT*, *GmLEA1‐Hap3*, and *GmLEC1‐Hap3* provides abundant genetic resources and a foundational reference for breeding soybeans with salt tolerance and high yield. The above results shift the focus from single‐gene to multi‐network breeding, optimizing trait performance through synergistic gene combinations and providing a new strategy for breeding salt‐tolerant crops.

### 3.4 Elite Salt‐Tolerant and High‐Yielding Soybean Hybrid Lines Developed via *GmPM30‐HapT* Introgression Using the KASP Marker

The superior *GmPM30* allele, particularly the salt‐tolerant and high‐grain‐yield *GmPM30‐HapT* allele, shows great potential for improving soybean salt tolerance. Therefore, we transferred *GmPM30‐HapT* into accessions carrying *GmPM30‐HapC* to develop inbred lines. To rapidly distinguish hybrid lines with different *GmPM30* alleles in breeding programs, we developed a KASP marker with 100% genotyping accuracy to differentiate between accessions carrying *GmPM30‐HapT* and *GmPM30‐HapC* (Figure 8a). Using this KASP marker, we then selected six elite accessions with high yields and strong salinity tolerance (Figure 8b). Among them, Hedou 12—a representative backbone soybean variety widely promoted and planted in Shandong Province—was suitable for use as a hybrid parent and exhibited a 36.7% higher grain weight per plant and a 25.5% higher 100‐seed weight than the average of all *GmPM30*‐*HapT* accessions under salinity stress. Under control conditions, Hedou 12 also showed 47.9% higher grain weight per plant and 40.4% higher 100‐seed weight than the soybean accessions with *GmPM30‐HapT* (Table S4, Supporting Information). To our knowledge, limited research has focused on molecular markers associated with soybean salt tolerance and yield‐related traits, as well as their applications in breeding. In general, the pyramiding of elite alleles in pursuit of higher crop yields during domestication or modern breeding programs may be accompanied by decreased frequency or even disappearance of alleles conferring stress tolerance. Our field trials in saline farmlands and greenhouse trials showed that molecular marker‐assisted rapid introduction of *GmPM30‐HapT* from Hedou 12 into the F_3_ hybrid lines resulted in improved salt tolerance and enhanced yield traits (Figure 8d–l). The grain yields under salt stress in these lines increased by approximately 20% compared to Hedou 12, providing a practical case for the application of this elite allele in breeding programs (Figure 8). Therefore, our study translates theoretical research into breeding practice by developing a high‐yielding and salt‐tolerant SNP marker and using it to generate elite hybrid lines. This suggests that molecular marker‐assisted breeding could be used to further pyramid three elite alleles within the GmLEA1‐GmPM30‐GmLEC1 interaction module, enabling rapid integration of multiple superior traits in future breeding programs.

Based on the findings of this study, we propose a possible model for the potential role of *GmPM30* in modulating salt tolerance in natural populations (Figure 7g). Elite alleles of *GmPM30* have undergone artificial selection during soybean domestication and breeding. At the protein level, the GmLEA1‐GmPM30‐GmLEC1 interaction module synergistically improves membrane stability and reduces oxidative damage. In parallel, at the transcriptional level, *GmPM30‐HapT* extensively and robustly activates the expression of genes involved in stress response pathways, thereby reducing oxidative stress. The integration of these two regulatory mechanisms ultimately conferred on *GmPM30‐HapT* with a significantly higher salt tolerance than *GmPM30‐HapC*.

## 4 Experimental Section

### Plant Materials and Growth Conditions

The 10 soybean (*Glycine max*) cultivars— Weidou 9 (C type), Williams 82 (T type), Qihuang 34 (T type), Liaodou14 (T type), Weidou 8 (T type), Dedou 99‐15 (T type), Jindou 34 (T type), Shanda 5 (T type), Hedou 18 (T type), and Hedou 12 (T type) were used for gene cloning, genetic transformation, and phenotypic assays. In the present study, all the soybean accessions were grown under short‐day conditions (8‐h light/16‐h dark photoperiod) in a climate room at 25 ± 1  C with a relative humidity of about 60%. For soybean hairy roots and transgenic lines growth phenotyping, salinity stress was imposed at the V3 growth stage (third trifoliate) and V1 growth stage by the addition of 150 mm NaCl to the water. To assay the salinity tolerance of soybean accessions during germination, soybean seeds were surface sterilized with chlorine gas; afterward, 10 seeds per genotype were placed in Petri dishes with filter paper soaked in water only (as control) or in 150 mm NaCl. The germination index was scored as described in a previous study.^[^ ^14^ ^]^ Each independent experiment included three technical replicate samples.

### Yield Related Traits Measurements and Field Experiments

The soybean accessions used for the haplotype phenotyping were planted at the two saline experimental farmlands, one of which was located in Dongying, China (37°19'N, 118°39'E), and the other was located in Weifang, China (36°41'N, 119°26'E). Field experiments of the bi‐parent genetic population from a cross of Jindou 28 × Hedou 12 were performed at Dongying, China (37°19'12′N, 118°39'43′E) was used for the validation of the genetic effect of different haplotypes of *GmPM30* on salinity tolerance. The experimental design was a randomized complete block design with three replicates. At all three sites, the row length was 1.5 m, the row spacing was 50 cm, and the plant spacing was 5 cm.

### Plasmid Construction and Plant Transformation

To generate the transgenic overexpression and RNA interference (RNAi) lines in soybean, the full‐length coding sequence of *GmPM30* was amplified from Weidou 9 complementary DNA (cDNA) and cloned into the Gateway pENTR/D‐TOPO vector with a Gateway pENTR/D‐TOPO Cloning Kit (Invitrogen by ThermoFisher Scientific, USA). The entry construct was then recombined into the destination binary vectors pB7WGF2 and pB7GWIWG2 (II) harboring the cauliflower mosaic virus (CaMV) 35S promoter in an LR reaction with LR Clonase mix. For RNAi of *GmPM30*, a 420‐bp specific fragment of the *GmPM30* coding sequence was cloned into the entry vector pENTR/D‐TOPO before an LR reaction was conducted with the binary vector pB7GWIWG2 (II). To construct the *GmPM30‐HapT* and *GmPM30‐HapC* overexpression plasmid, the CDS of *GmPM30‐HapT* and *GmPM30‐HapC* were ligated into pTF101 with the restriction sites BamHI and SacI and then transformed into Qihuang 34. To generate CRISPR/Cas9‐mediated mutations, four guide RNA (gRNA) targets were designed using CRISPR‐P v2.0 (http://crispr.hzau.edu.cn/CRISPR2/) and transferred to the CRISPR/Cas9 vector (pGES402). The resulting constructs were introduced into *Agrobacterium* (*Agrobacterium tumefaciens*) strain GV3101 and EHA105, and *Agrobacterium rhizogenes* strain K599 after sequence verification. To obtain *gmpm30‐ko* transgenic hairy roots, RFP fluorescence screening was first performed using a LUYOR‐3415RG dual‐fluorescent observation system to select positive hairy roots. A subset of positive hairy roots was then subjected to PCR amplification and sequencing to verify editing types, confirming the generation of independent knockout mutant lines. Primer information is shown in Table S1 (Supporting Information).

### RNA Isolation, cDNA Preparation, and Reverse Transcription Quantitative PCR Assays

TRIgent (Mei5bio) was used to extract total RNA from different soybean tissues. Reverse transcription was performed using an EVO M‐MLV RT with gDNA Clean for qPCR II kit (ACCURATE BIOLOGY) according to the manufacturer's instructions. MonAmp ChemoHS qPCR Mix (Monad) was used for quantitative PCR (qPCR) on a QuantStudio 1 instrument (Thermo Fisher Scientific) following the manufacturer's instructions. Relative expression levels were calculated using the 2^−ΔΔCT^ method. *GmELF1b* was used as the internal control. Three biological replicates and three technical replicates were performed.

### Subcellular Localization Assay

The full‐length *GmPM30* coding sequence without the stop codon was cloned into the GFP‐pB7WGF2 plant expression vector to produce the *35Spro: GFP‐GmPM30* construct. The resulting construct was introduced into Agrobacterium strain GV3101 before being infiltrated into the leaves of 4‐week‐old *Nicotiana benthamiana* plants via Agrobacterium‐mediated infiltration. The detection of green (excitation, 488 nm; emission, 520 ± 15 nm) and red fluorescence signals (excitation, 532 nm; emission, 570 ± 15 nm) was carried out by laser scanning confocal microscopy (LSM 880; Carl Zeiss, Oberkochen, Germany).

Yeast two‐hybrid (Y2H), bimolecular fluorescence complementation (BiFC), luciferase complementation imaging (LCI), and Co‐immunoprecipitation (Co‐IP) assays: For the yeast two‐hybrid assays, the full‐length coding sequence of GmPM30 or GmLEC1 and GmLEA1 without the stop codon were introduced into the bait vector (pGBKT7) or prey vector (pGADT7), respectively. The resulting construct GmPM30‐pGBKT7 was co‐transformed with GmLEC1‐pGADT7 or GmLEA1‐pGADT7 into yeast strain Y2HGold. Positive transformants were selected on double‐dropout medium lacking Leu and Trp (DDO); protein–protein interactions were tested on quadruple‐dropout medium lacking Leu, Trp, His, and Ade (QDO) with growth at 30 °C for 2–4 days. For the construction of BiFC vectors, the same GmPM30 coding sequence was cloned into pX‐nYFP to generate GmPM30‐nYFP. The full‐length coding sequences of GmLEC1 and GmLEA1 without the stop codon were individually cloned into pX‐cYFP to generate GmLEC1‐cYFP and GmLEA1‐cYFP. For the construction of LUC vectors, the CDS of GmPM30, GmLEA1, and GmLEC1 were cloned into the pCAMBIA1300‐nLUC/cLUC vectors to generate GmPM30‐HapT/HapC‐nLUC, GmLEA1‐nLUC, GmLEC1‐nLUC, cLUC‐GmLEA1, cLUC‐GmLEC1, and cLUC‐ GmPM30‐HapT/HapC constructs, which were used for firefly LCI assays. All constructs were individually transformed into the Agrobacterium strain GV3101 before being co‐infiltrated as appropriate pairs into N. benthamiana leaves and cultivated at 25°C for ≈48 h. The detection of the yellow fluorescence signal was carried out using a laser scanning confocal microscope (LSM 880; Carl Zeiss, Oberkochen, Germany). The CDS of GmLEA1 and GmLEC1 were cloned into the pCAMBIA3300 to generate GmLEA1‐mCherry and GmLEC1‐mCherry. The GFP‐GmPM30‐HapT/HapC, GmLEA1‐mCherry, GmLEC1‐mCherry, or GFP constructs were transformed into Agrobacterium GV3101, and Agrobacteria harboring the indicated constructs were co‐infiltrated into the N. benthamiana leaves. The plants were grown for 40h at 25 °C before the infiltrated leaves were collected. The total protein extraction and Co‐IP assays were performed using the method in a previous study.^[^ ^35^ ^]^ The anti‐mCherry (Proteintech, Chicago, USA) or anti‐GFP (Proteintech, Chicago, USA) antibodies were used for immunoblotting analysis. The primers used in the generation of the relevant construct are listed in Table S1 (Supporting Information).

### Haplotype Analysis of GmPM30, GmLEA1, and GmLEC1 in the Soybean Population

Using the Williams 82 genome as a reference, the genome sequence covering a 1.5‐kb promoter fragment and the full length of *GmPM30*, *GmLEA1*, and *GmLEC1* were selected in resequencing data from 555 soybean accessions. The geographical distribution of *GmPM30* was surveyed using soybean cultivars collected from 22 regions in northern China (Heilongjiang, Jilin, Liaoning, Shanxi, Neimenggu, Xinjiang, and Gansu provinces), Huanghuai region (Anhui, Hebei, Henan, and Shandong provinces), and southern China (Fujian, Guangdong, Guangxi, Guizhou, Hunan, Hubei, Jiangsu, Jiangxi, Sichuan, Yunnan, and Zhejiang provinces). The high‐quality SNPs were used to establish haplotype networks with Haploview software.^[^ ^54^ ^]^ Gene‐based association analysis was performed in Tassel 5.0 using a mixed linear model.^[^ ^55^ ^]^

### Selection and Evolutionary Analysis

The population differentiation statistics (F_ST_), π values (nucleotide diversity), and Tajima's D of the different subpopulations were calculated by using VCFtools software.^[^ ^56^ ^]^ The 5000‐kb windows and 100‐kb steps were used for population genetics analysis.

### Transcriptome Analysis

For the transcriptome analysis, TRIgent (Mei5bio) was used to extract RNA from the soybean hairy root material of 3 biological replicates of EV, *GmPM30‐HapT*‐OE, and *GmPM30‐HapC*‐OE transgenic hairy roots under normal and salt‐stressed conditions. The cDNA libraries were sequenced on the Illumina sequencing platform by Genedenovo Biotechnology Co., Ltd (Guangzhou, China). After sequencing, low‐quality raw reads were filtered, and then high‐quality clean reads were mapped to the soybean reference *Glycine max* Wm82.a2. v1 genome. Gene expression was calculated and normalized to TPM (Transcripts Per Kilobase per Million) values. DESeq2 was used to identify the DEGs (log2 fold change ≥1 and q‐value < 0.05). Enrichment analysis of the GO and KEGG of the DEGs was performed by Omicsmart (https://www.omicsmart.com).

### Enzyme Activity and Malondialdehyde Content Determination

Malondialdehyde (MDA) concentration (Nanjing Jiancheng Bioengineering Institute, China, Cat: A003‐3‐1), peroxidase (POD) activity (Nanjing Jiancheng Bioengineering Institute, China, Cat: A084‐3‐1), and superoxide dismutase (SOD) activity were measured using specific detection kits (Beyotime, China, Cat: S0109), following the manufacturer's instructions.

### Quantification of H_2_O_2_ Contents

The H_2_O_2_ contents were determined using a hydrogen peroxide (H_2_O_2_) content assay kit (Solarbio, China), following the manufacturer's instructions. Three or more biological replicates were included for each assay.

### Measurement of Ion Leakage

For membrane ion leakage measurement, *N. benthamiana* leaves of the same size were picked from the single infection and co‐infiltrated cell suspensions under normal and salt stress treatments. The leaves were collected in a 10 mL EP tube with 4 mL of deionized water and oscillated for 2h, and the initial electrical conductivity (C1) was measured with an electro‐conductivity meter. And then the electrical conductivity (C2) was measured after boiling for 15 min and cooled to room temperature with an electro‐conductivity meter again. The ion leakage (C) was calculated as the ratio of C1/C2.

### KASP Marker Development for Genotyping of Different Haplotypes

A series of high‐concentration (>20 ng µL^−1^) and high‐quality genomic DNA samples from soybean cultivars were obtained by CTAB for KASP marker‐based genotyping. A 300‐bp fragment containing 150 bp before and 150 bp after the polymorphic site in the coding region was selected to design KASP primers with Primer 5.0 software. The KASP assay was performed in a 96‐well plate on a CFX Connect Real‐Time System (Bio‐Rad, USA) following the instructions listed in Table S1 (Supporting Information).

### Statistical Analysis

All statistical analyses were performed using GraphPad Prism software version 8.0. Significant differences were analyzed using one‐tailed, two‐sample Student's *t*‐tests or two‐way analysis of variance (ANOVA). Details of the statistical analyses were given in the figure legends.

## Conflict of Interest

The authors declare no conflict of interest.

## Author Contributions

F.N.X. conceived the project. S.Y.H., F.N.X., Q.L., and S.L. designed the experiments. S.Y.H. drafted the manuscript and performed most of the experiments. Y.H.X., J.T.Y. and W.Y.Z. performed transformation experiments. X.J.Z. provided the germplasm resources. T.S.L., Y.J.S., X.C., and H.Z. provided the phenotype of soybean germplasm resources. Y.H.X., X.C., and Z.J.Z. performed parts of the biochemical and phenotyping experiments. F.N.X. and Q.L. revised the manuscript. All authors read and approved of its content.

## Supporting information



Supporting Information

Supporting Information

Supporting Information

## Data Availability

The raw sequence data reported in this paper have been deposited in the Genome Sequence Archive in National Genomics Data Center (CNCB‐NGDC Members and Partners), China National Center for Bioinformation/Beijing Institute of Genomics, Chinese Academy of Sciences (GSA: CRA018960 and GSA: CRA025798), which are publicly accessible at https://ngdc.cncb.ac.cn/gsa/s/t1wa9N34 and https://ngdc.cncb.ac.cn/gsa/s/9gs9E9lD, respectively.
