# Supplementary material for: A Coding SNP in GmPM30 Enhances Soybean Salinity Tolerance and Yield through the GmLEA1‐GmPM30‐GmLEC1 Module
Source: Adv Sci (Weinh). 2025 Oct 6;12(44):e09391. doi: 10.1002/advs.202509391 (PMC12667475; doi:10.1002/advs.202509391)
Supplement: Supplementary file 1 — Supporting Information [file ADVS-12-e09391-s001.docx]

**A Coding SNP in *GmPM30* Enhances Soybean Salinity Tolerance and Yield through the GmLEA1-GmPM30-GmLEC1 Module**

*Shiyu Huang, Yuhan Xia, Jingting Yang, Yujun Si, Xue Chen, Hao Zhang, Tianshi Liu, Wenyu Zheng, Xin Chen, Zhongjuan Zhao, Xiaojian Zheng, Qing Lu^*^, Shuo Li^*^, Fengning Xiang^*^*

S. Huang, Y. Xia, J. Yang, T. Liu, W. Zheng, X. Chen, Z. Zhao, X. Zheng, Q. Lu, S. Li, F. Xiang

The Key Laboratory of Plant Development and Environmental Adaptation Biology, Ministry of Education

Shandong Key Laboratory of Precision Molecular Crop Design and Breeding

School of Life Sciences

Shandong University

Qingdao 266237, China

E-mail [qing.lu@sdu.edu.cn](mailto:qing.lu@sdu.edu.cn), [lishuo@sdu.edu.cn](mailto:lishuo@sdu.edu.cn), [xfn0990@sdu.edu.cn](mailto:xfn0990@sdu.edu.cn)

Yu. Si, X. Chen, H. Zhang

Weifang Academy of Agricultural Sciences

Weifang, 261071, China.


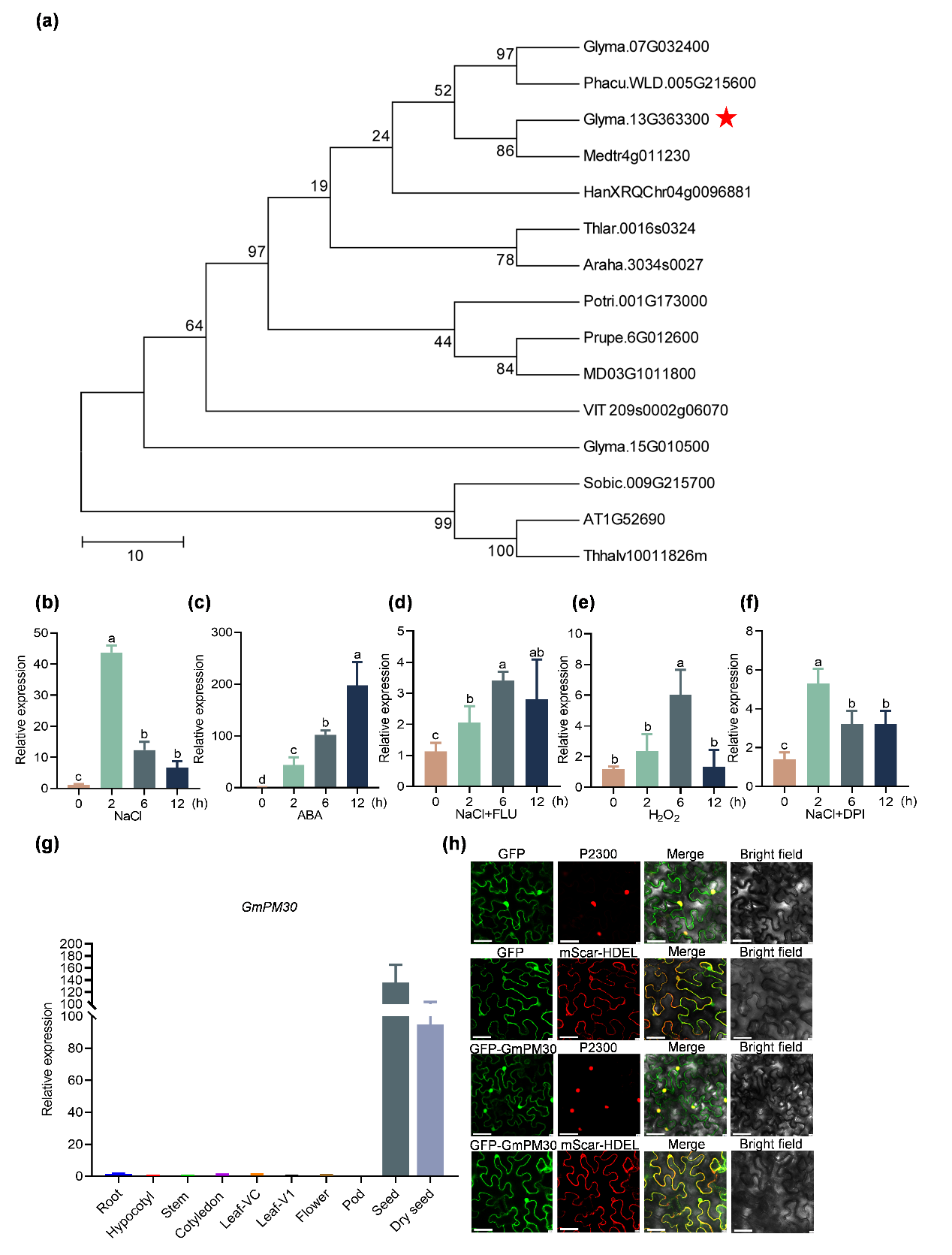


**Supplemental Figure 1. Characterization of *GmPM30.***

**(a)** Phylogenetic tree of *GmPM30* and its homologs from different plant species, reconstructed with the neighbor-joining method with 1000 bootstrap replicates in MEGA 7. (**b)** Relative *GmPM30* expression levels in response to treatment with 150 mM NaCl in the soybean cultivar Williams 82. (**c)** Relative *GmPM30* expression levels in response to treatment with 100 μM ABA in the soybean cultivar Williams 82. (**d)** Relative *GmPM30* expression levels in response to treatment with 150 mM NaCl and 50 nM fluridone (FLU) in the soybean cultivar Williams 82. **(e)** Relative *GmPM30* expression levels in response to treatment with 10 mM H_2_O_2_ in the soybean cultivar Williams 82. **(f)** Relative *GmPM30* expression levels in response to treatment with 150 mM NaCl and 100 µM diphenyliodonium chloride (DPI) in the soybean cultivar Williams 82. (**g)** Expression pattern of *GmPM30* in different tissues in the soybean cultivar Williams 82. Data are means ± standard deviation (SD) from three biological replicates. **(h)** Subcellular localization of GmPM30 in *N. benthamiana* epidermal cells. Nucleus were marked with the P2300; the endoplasmic reticulum (ER) was marked with the *pCambia-mScarlet-HDEL*. Scale bar, 50 μm.


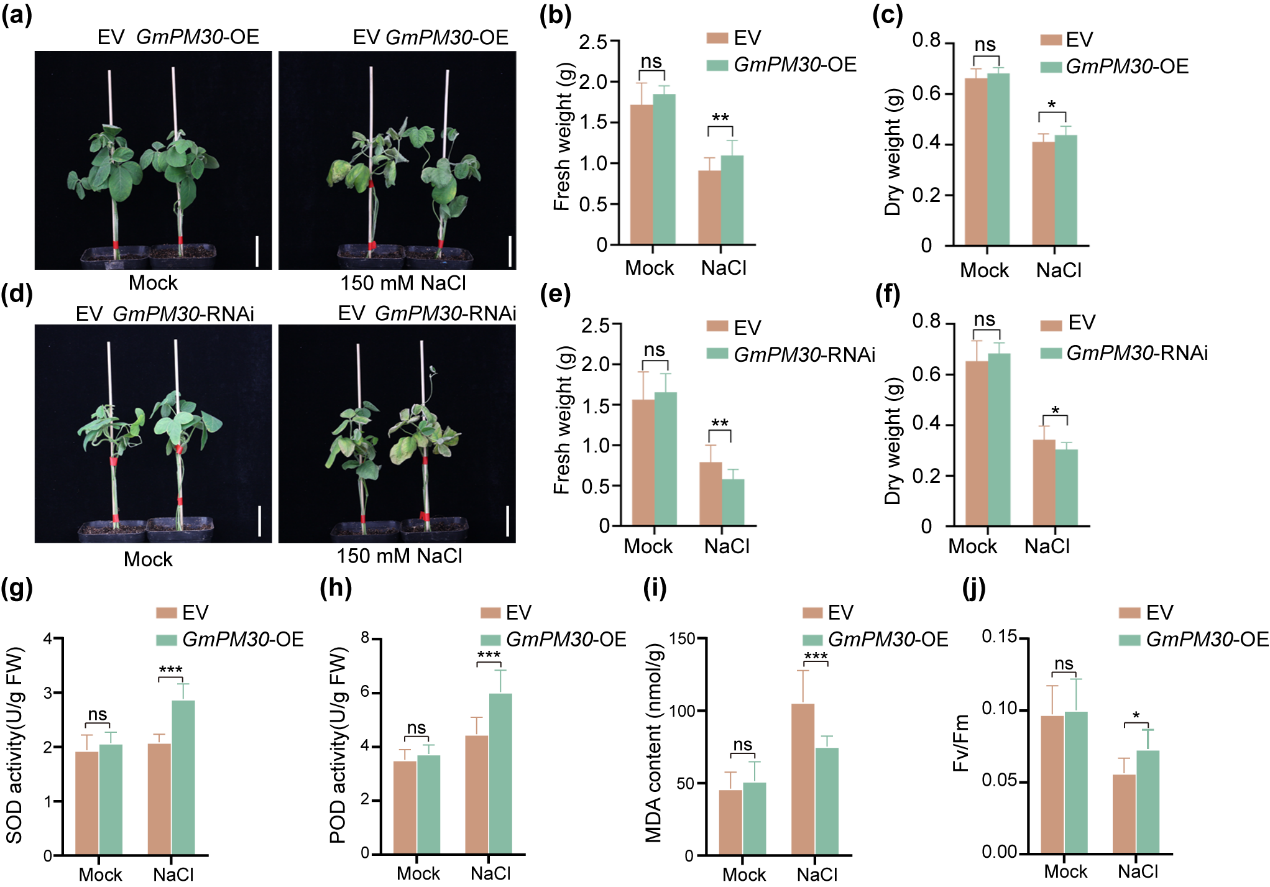


**Supplemental Figure 2. *GmPM30* enhances salt tolerance in soybean hairy roots.**

**(a, d)** Representative images of control cultivar Williams 82 (transformed with empty vector as control, EV), *GmPM30*-OE, and *GmPM30*-RNAi soil-grown plants at the vegetative stage under mock (H_2_O only) or 150 mM NaCl treatment, taken 7 days after the onset of salinity treatment. Scale bars, 5 cm. **(b, e)** Quantification of fresh weight from control (EV), *GmPM30*-OE (**b**), and *GmPM30*-RNAi (**e**) transgenic hairy roots. **(c, f)** Quantification of dry weight from control (EV), *GmPM30*-OE (**c**), and *GmPM30*-RNAi (**f**) transgenic hairy roots. **(g–j)** Superoxide dismutase (SOD) activity (**g)**, Peroxidase (POD) activity (**h),** malondialdehyde (MDA) content **(i),** and *Fv/Fm* (**j**) of control (EV) and *GmPM30*-OE transgenic hairy roots under control and 150 mM salt treatment. Data are means ± SD from 10 biological replicates (two-way ANOVA with **P* < 0.05).


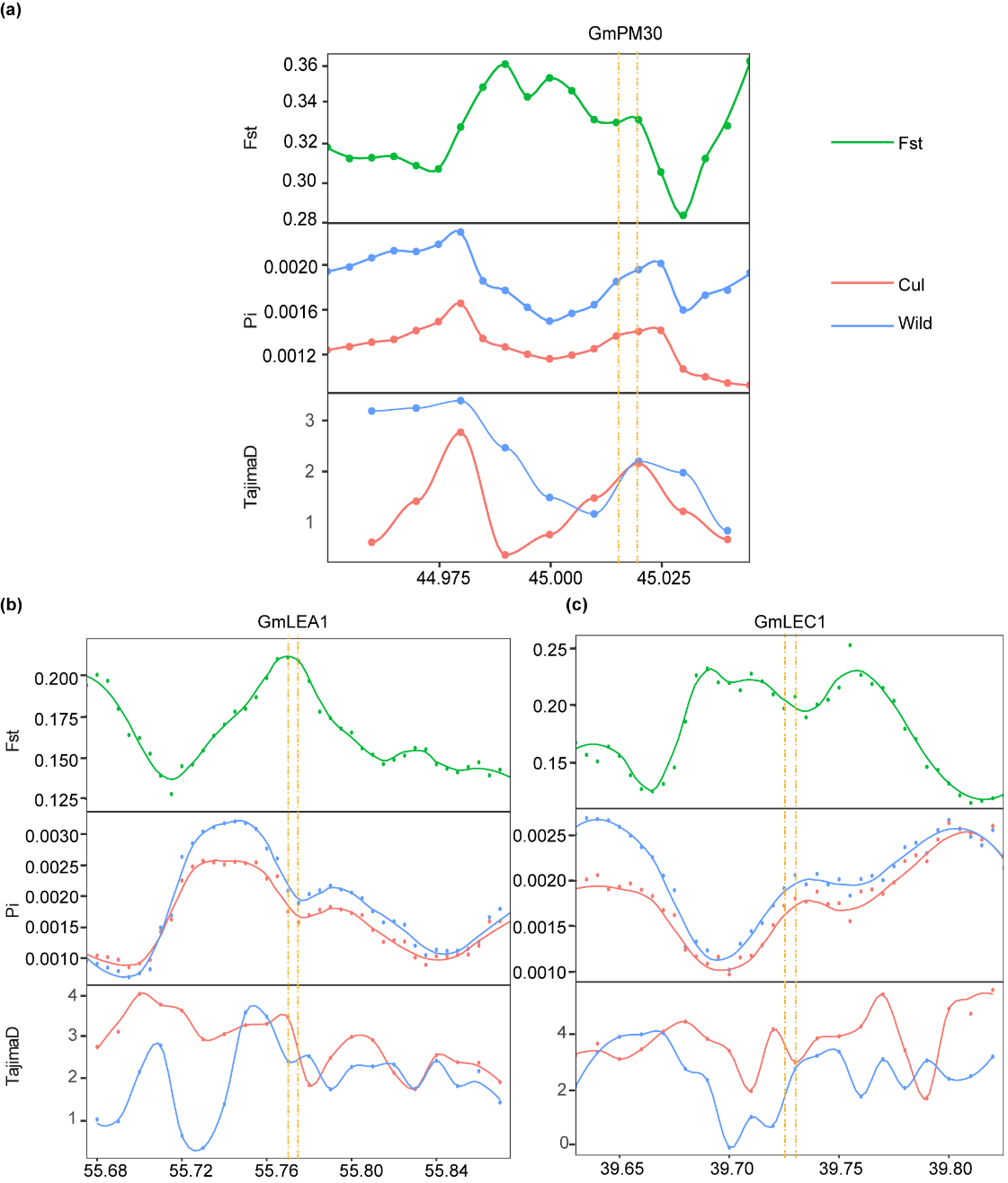


**Supplemental Figure 3. Selective sweep analysis of *GmPM30*, *GmLEA1* and *GmLEC1.***

*F_ST_*, nucleotide diversity, and Tajima’D values over the genomic region containing the *GmPM30* locus among wild, landrace, and soybean germplasms.


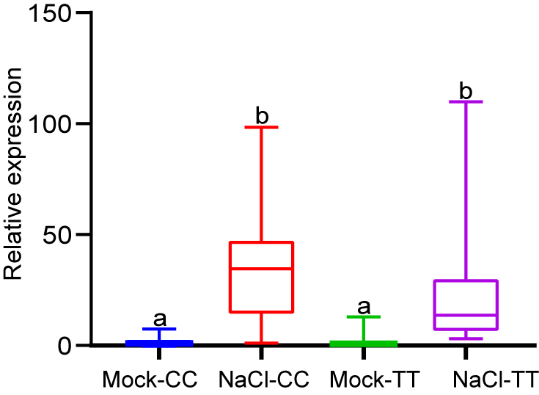


**Supplemental Figure 4. Relative *GmPM30* expression levels as determined by RT-qPCR in *HapT* and *HapC* soybean accessions with or without salinity stress.**

Relative *GmPM30* expression in soybean accessions harboring the *HapT* or *HapC* allele of *GmPM30*. These plants grew in soil and watered with water only or 150 mM NaCl Different lowercase letters indicate significant differences.


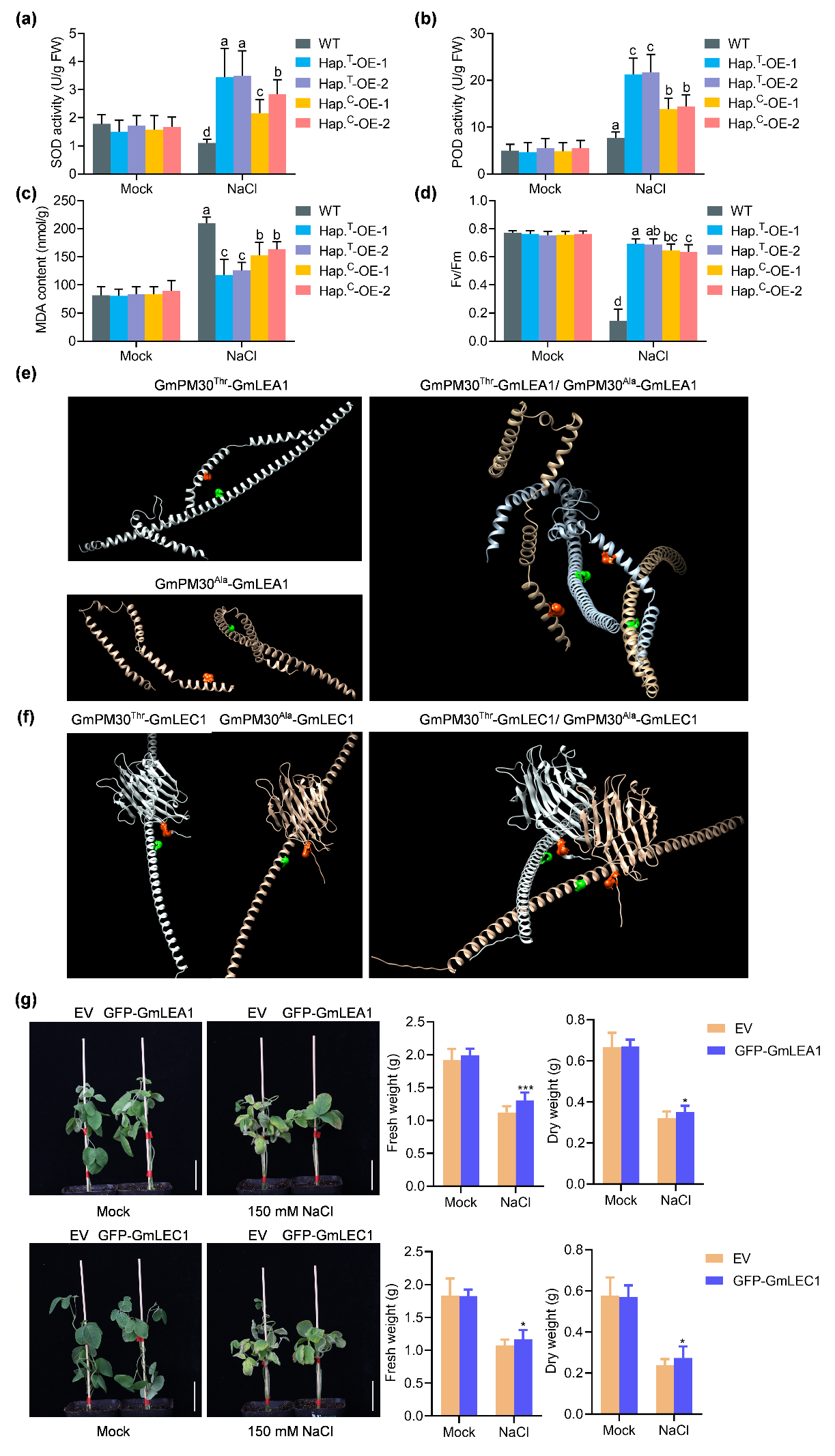


**Supplemental Figure 5. *GmPM30-HapT* had stronger salt tolerance than *GmPM30-HapC* in soybean plants.**

**(a-d)** Superoxide dismutase (SOD) activity **(a)**, Peroxidase (POD) activity **(b),** malondialdehyde (MDA) content **(c),** and *Fv/Fm* **(d)** of WT, *HapT*-OE and *HapC*-OE plants under control and 150 mM salt treatment. Data are means ± SD from 10 biological replicates (two-way ANOVA with **P* < 0.05). **(e-f) S**tructural predictions of GmPM30^Thr^/GmPM30^Ala^-GmLEA1 **(e)** and GmPM30^Thr^/GmPM30^Ala^-GmLEC1 **(f)**. In the silver protein structure, green indicates Thr; in the yellow protein structure, green indicates Ala; and red indicates the interaction site between the four groups of proteins. **(g)** Representative images of soil-grown control (EV) and *GmLEA1*-OE and *GmLEC1*-OE plants at the vegetative stage under mock treatment (H_2_O) or 150 mM NaCl treatment. Fresh weight and dry weight of control (EV) and *GmLEA1*-OE and *GmLEC1*-OE transgenic hairy roots under mock treatment (H_2_O) and 150 mM salt treatment. Data are means ± SD from 10 biological replicates. Scale bars, 6 cm.


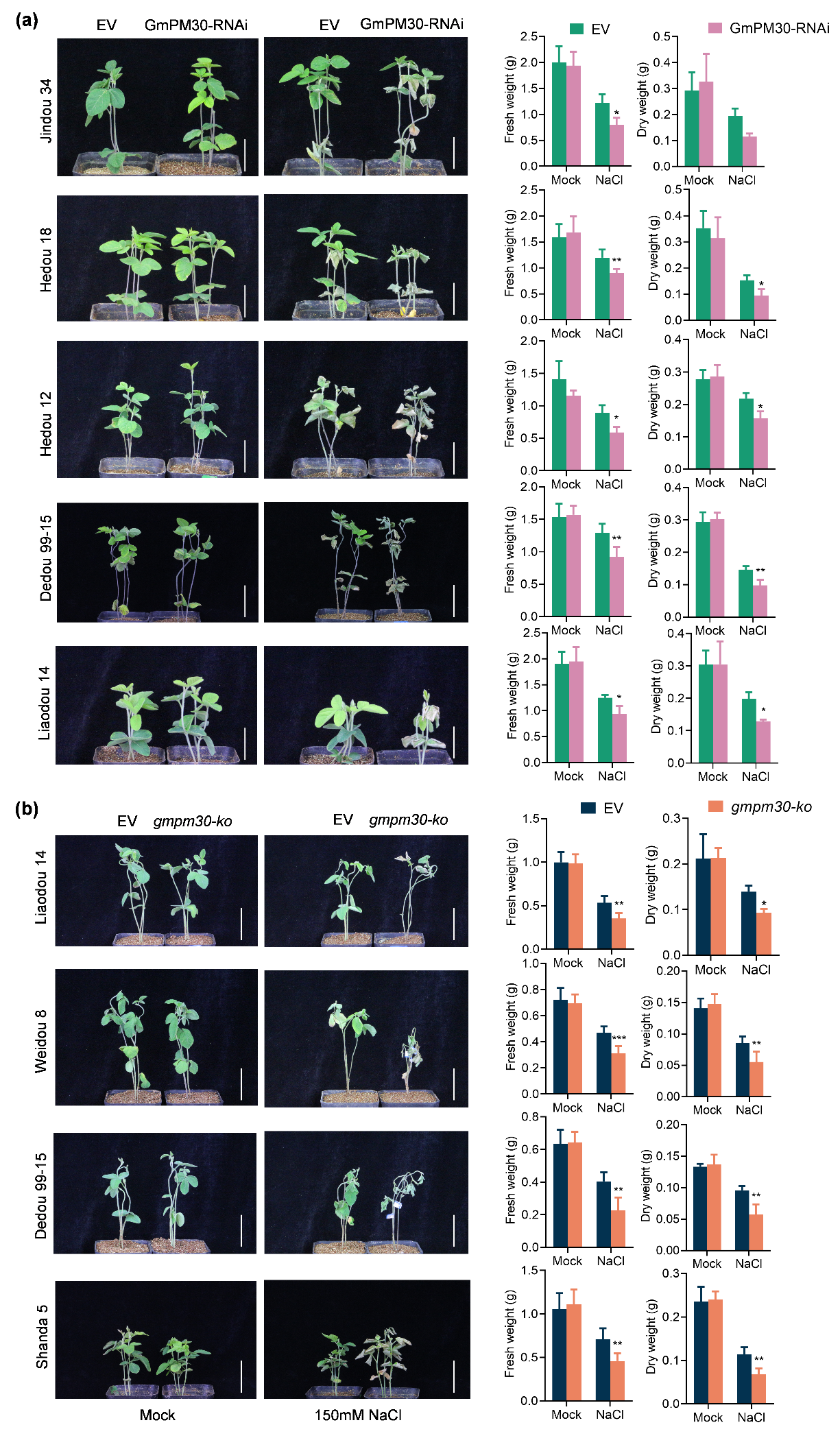


**Supplemental Figure 6. Functional verification of *GmPM30* in the soybean hairy root system using cultivars containing *HapT.***

**(a)** Representative images and biomass of soil-grown control (EV) and GmPM30-RNAi plants at the vegetative stage under mock treatment (H_2_O) or 150 mM NaCl treatment in five cultivars containing *HapT* (Jindou 34, Hedou 18, Hedou12, Dedou 99-15 and Liaodou 14). **(b)** Representative images and biomass of soil-grown control (EV) and *gmpm30-ko* plants at the vegetative stage under mock treatment (H_2_O) or 150 mM NaCl treatment in four cultivars containing *HapT* (Liaodou 14, Weidou 8, Dedou 99-15 and Shanda 5). Data are means ± SD from 10 biological replicates. Scale bars, 4 cm.


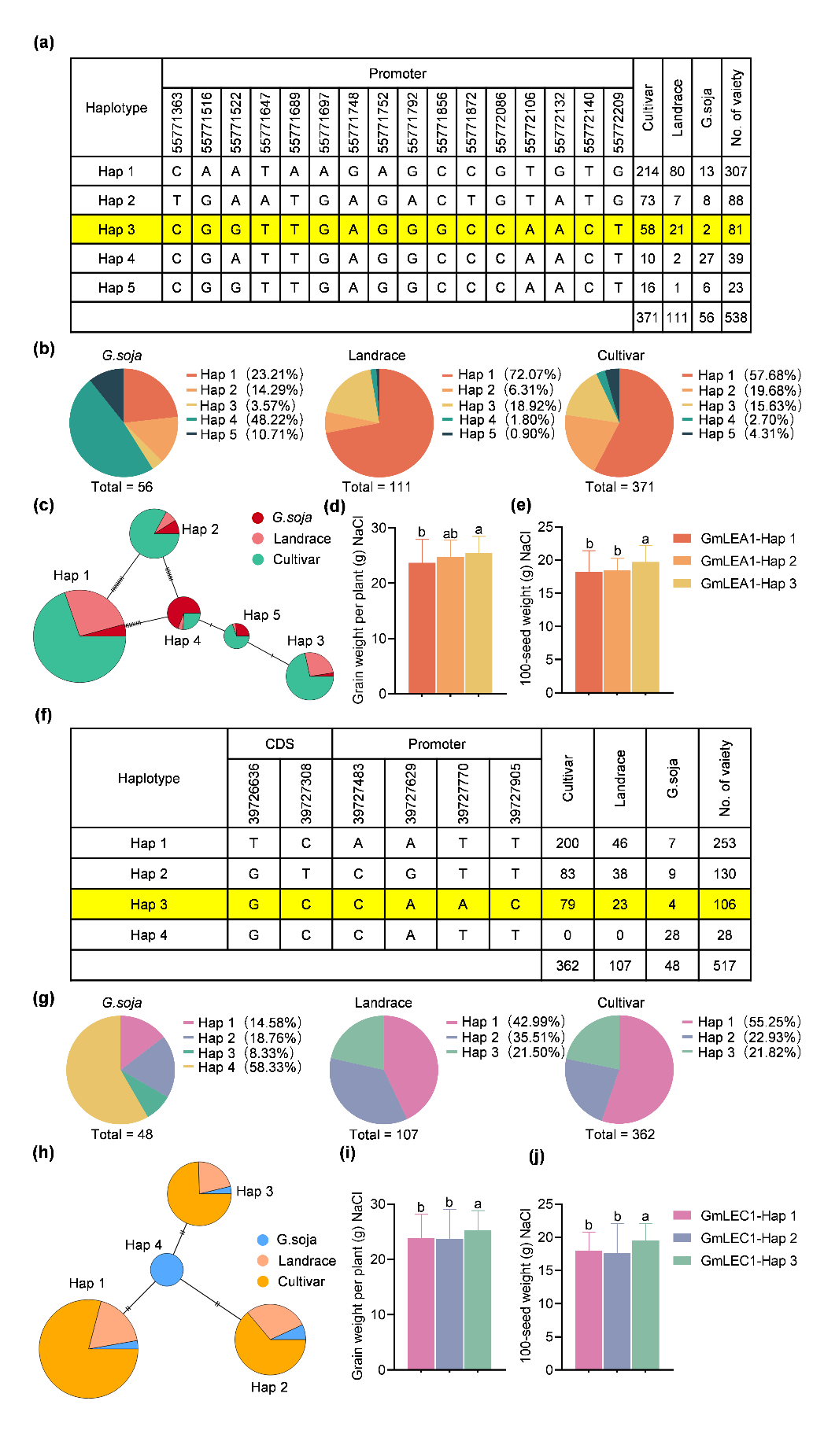


**Supplemental Figure 7. Haplotype analysis of *GmLEA1* and *GmLEC1* in Chinese soybean accessions.**

**(a, f)** Summary of *GmLEA1* **(a)** and *GmLEC1* **(f)** haplotypes detected in natural populations. The number of varieties for each haplotype (*Hap1–5*) and (*Hap1–4*) is shown to the right. **(b, g)** Distribution of the five main haplotypes at *GmLEA1* **(b)** and four main haplotypes at *GmLEC1* **(g)** in wild soybeans, landraces, and improved cultivars. **(c, h)** Haplotype network analysis. Circle size is proportional to the number of accessions harboring each haplotype, while circle colors represent the different soybean groups. **(d-e, i-j)** Summary of the two grain yield traits for plants in saline soils with the different haplotypes of *GmLEA1* **(d-e)** and *GmLEC1* **(i-j)**.


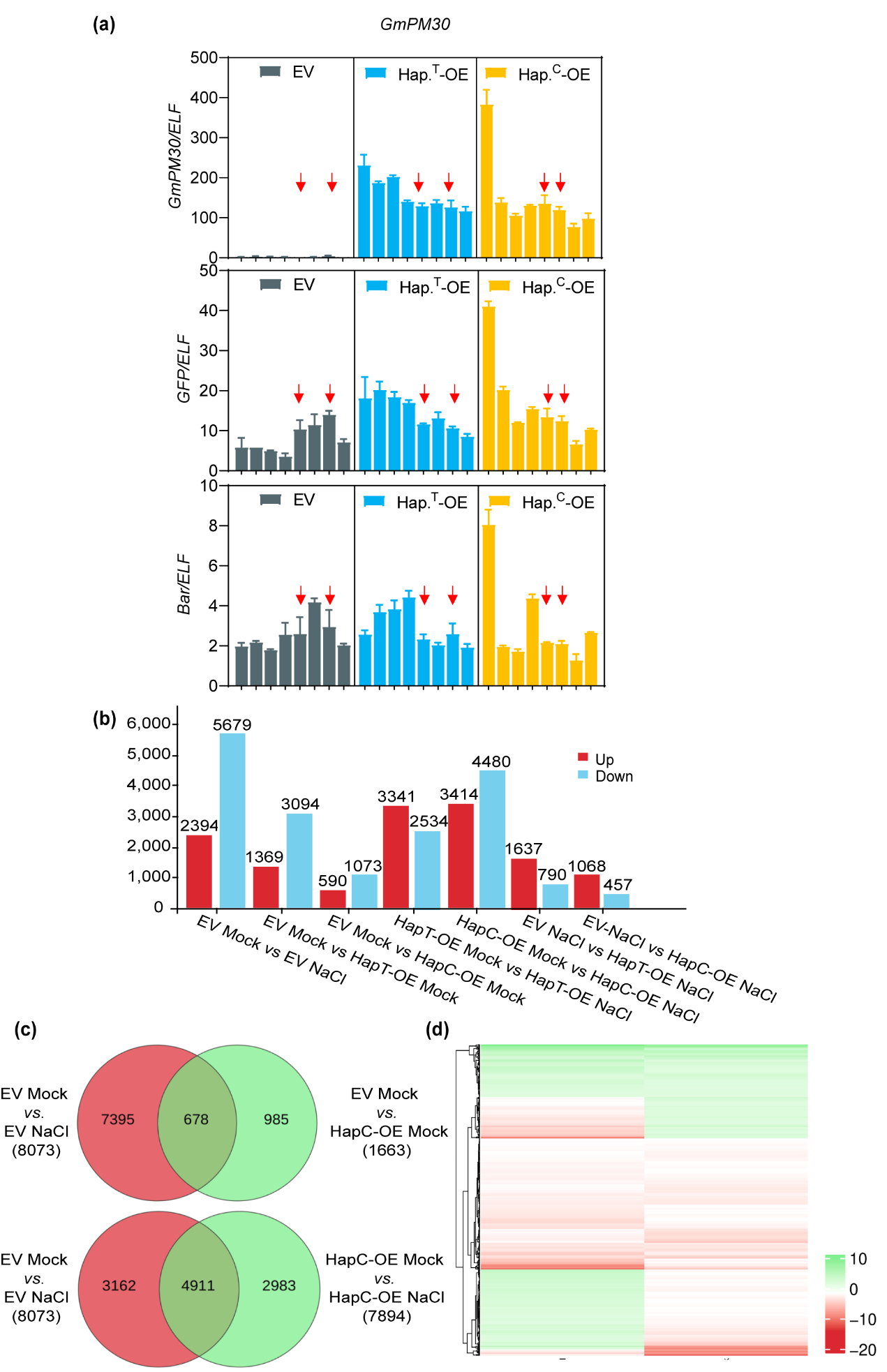


**Supplemental Figure 8. RNA-seq analysis of *HapT*-OE, *HapC*-OE and EV transgenic soybean hairy roots.**

**(a)** Expression of *GmPM30*, *GFP* and *Bar* in EV, *HapT*-OE and *HapC*-OE soybean hairy roots. **(b)** Number of upregulated or downregulated differentially expressed genes

(DEGs) from the comparisons of EV *vs.* *HapT* *vs*. *HapC* under mock and salt stress conditions. **(c)** Venn diagrams showing the extent of overlap for DEGs between *HapC*-OE and EV transgenic soybean hairy roots under mock and salinity stress conditions. **(d)** Hierarchical cluster analysis of the overlapping genes from **(c)** differentially expressed in mock-treated EV versus NaCl-treated EV and mock-treated EV versus mock-treated *HapC*-OE. The numerical values in the gradient bar represent log2 (fold change) relative to the control sample.


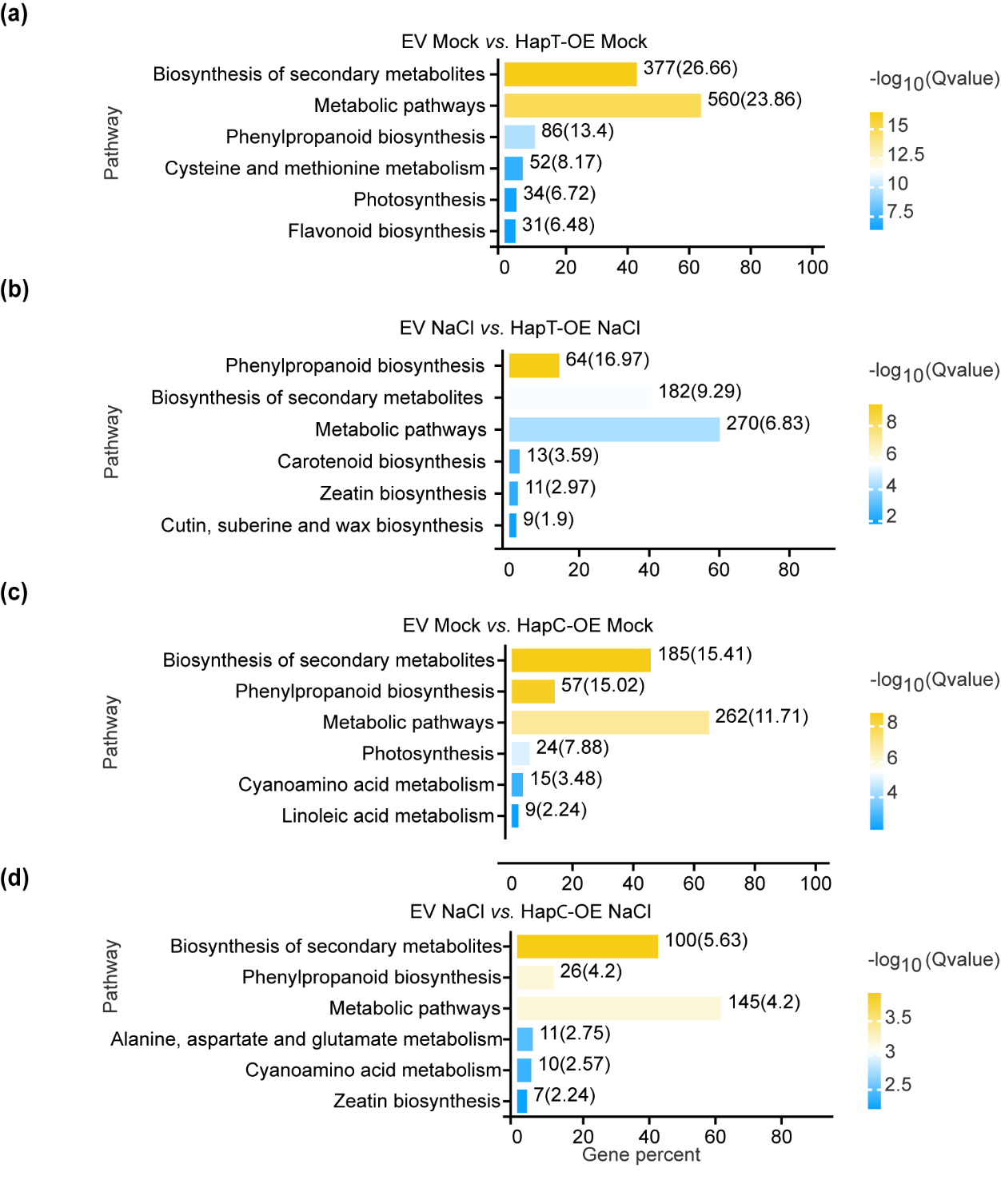


**Supplemental Figure 9. KEGG enrichment analysis of *HapT*-OE, *HapC*-OE and EV transgenic soybean hairy roots under mock and salt conditions**

**(a-b)** Significantly enriched pathways among DEGs in EV mock *vs.* *HapT*-OE mock and EV NaCl vs *HapT*-OE NaCl. **(c-d)** Significantly enriched pathways among DEGs in EV mock *vs.* *HapC*-OE mock and EV NaCl vs *HapC*-OE NaCl. Numbers near columns indicate the number of DEGs with corresponding annotation and the q-value.


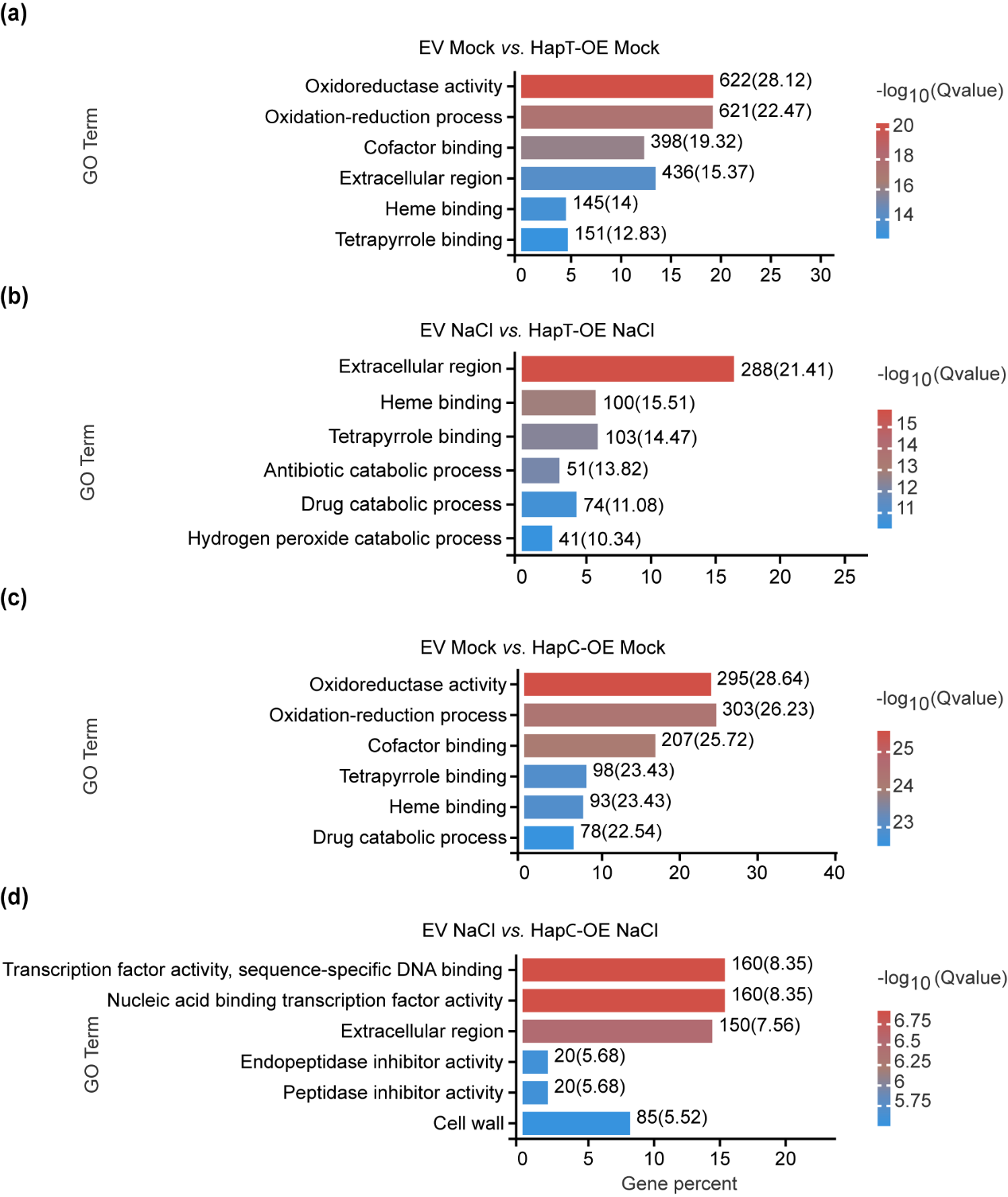


**Supplemental Figure 10. GO enrichment analysis of *HapT*-OE, *HapC*-OE and EV transgenic soybean hairy roots under mock and salt conditions.**

**(a-b)** Significantly enriched pathways among DEGs in EV mock *vs.* *HapT*-OE mock and EV NaCl vs *HapT*-OE NaCl. **(c-d)** Significantly enriched pathways among DEGs in EV mock *vs.* *HapC*-OE mock and EV NaCl vs *HapC*-OE NaCl. Numbers near columns indicate the number of DEGs with corresponding annotation and the q-value.


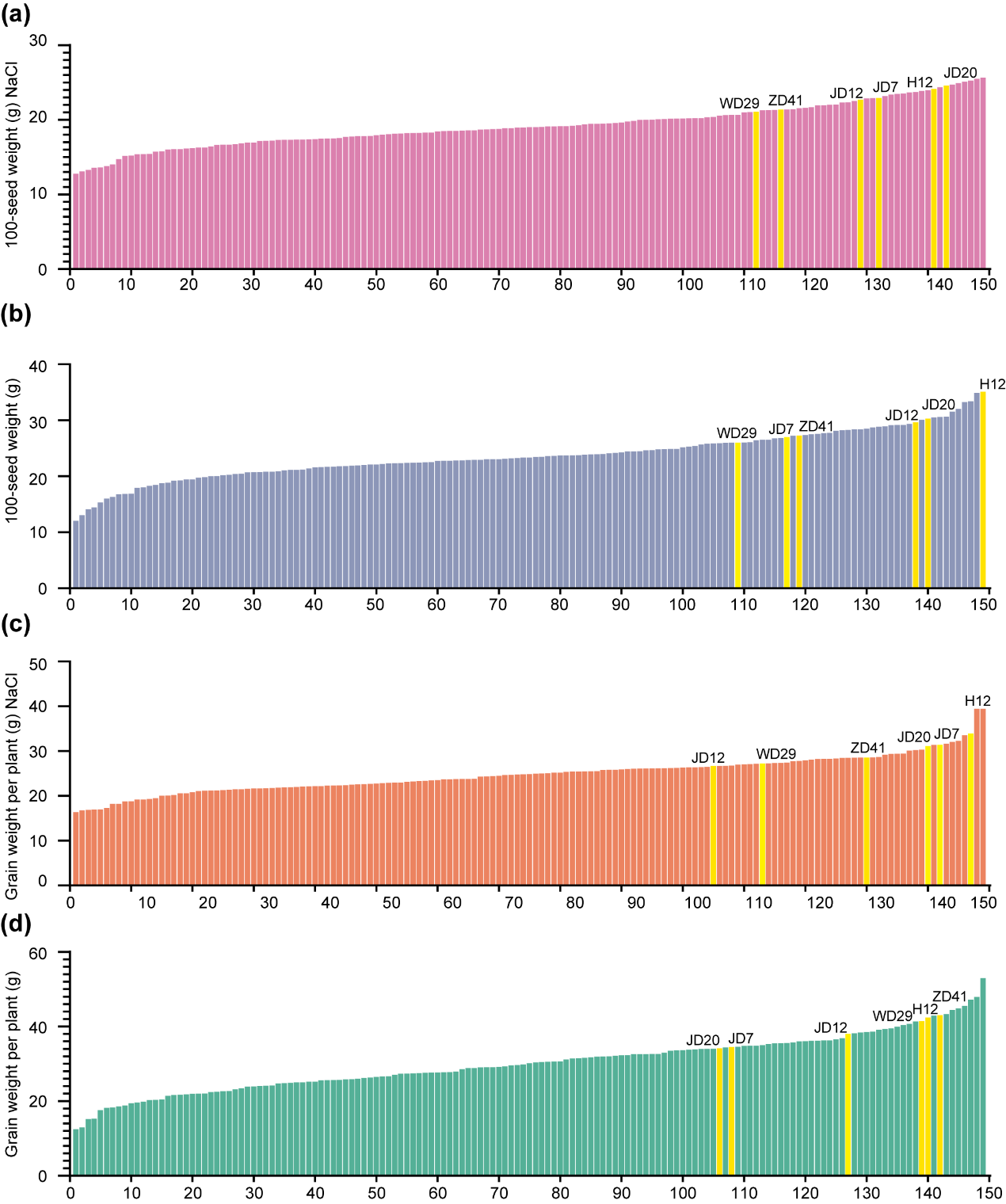


**Supplemental Figure 11. Yield traits under control and salinity stress conditions in HapT soybean accessions.**

Summary of the 100 seed weight **(a-b)** and grain weight per plant **(c-d)** for soybean accessions harboring *HapT* in saline or normal soils. All yield-related traits were ranked from small to large, and the yellow rectangles represent six elite donor accessions selected by the KASP marker designed to distinguish between *HapC* and *HapT* of *GmPM30*. WD 29, Wandou 29; ZD 41, Zhongdou 41; JD 12, Jidou 12; JD 7, Jidou 7; H12, Hedou 12; JD 20, Jidou 20.
