# Supplementary figures and images for: A Coding SNP in GmPM30 Enhances Soybean Salinity Tolerance and Yield through the GmLEA1‐GmPM30‐GmLEC1 Module
Source: Adv Sci (Weinh). 2025 Oct 6;12(44):e09391. doi: 10.1002/advs.202509391 (PMC12667475; doi:10.1002/advs.202509391)

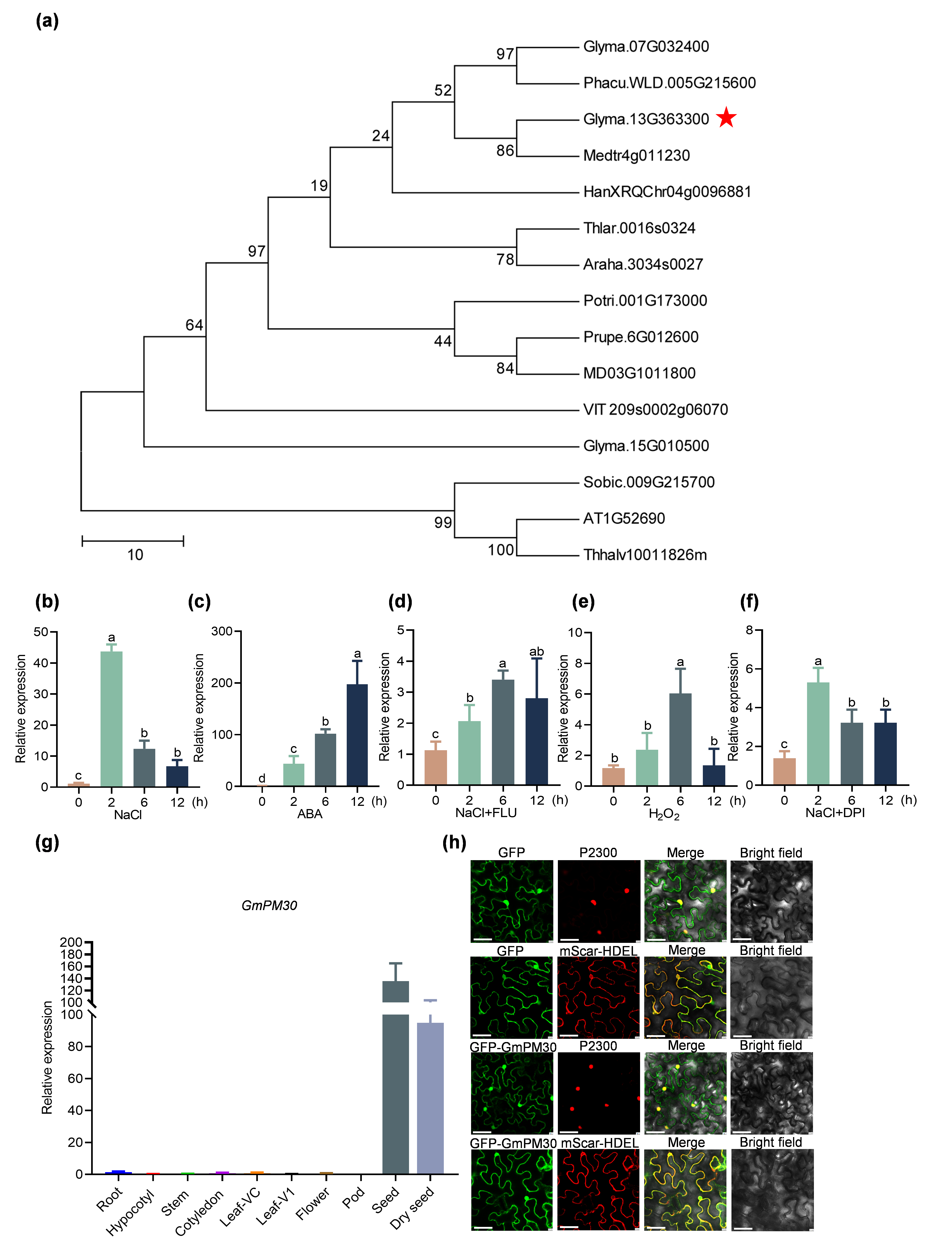

Supplement: Supplementary file 2 — Supporting Information [file ADVS-12-e09391-s003.zip › Figure-S1.tif]

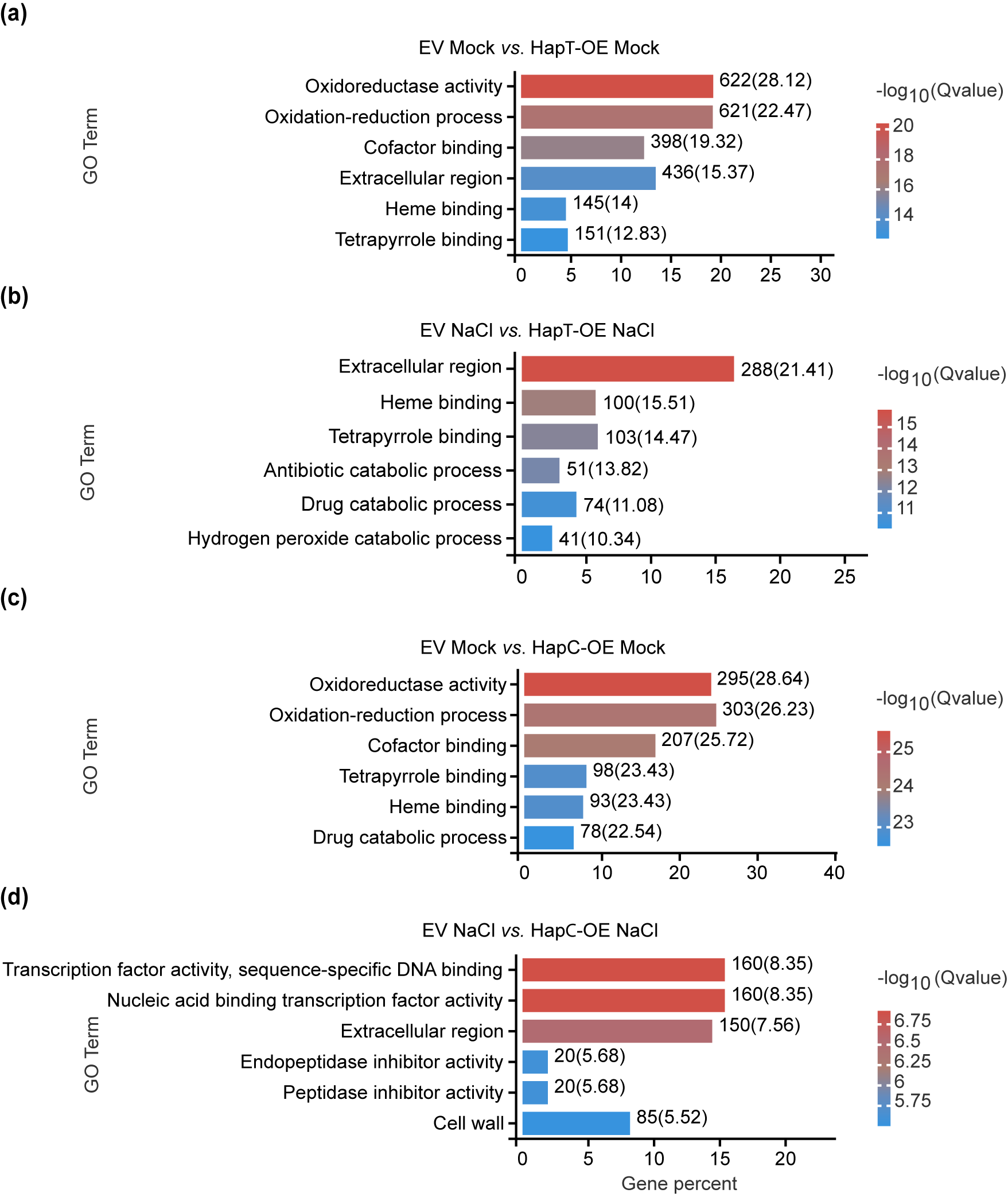

Supplement: Supplementary file 2 — Supporting Information [file ADVS-12-e09391-s003.zip › Figure-S10.tif]

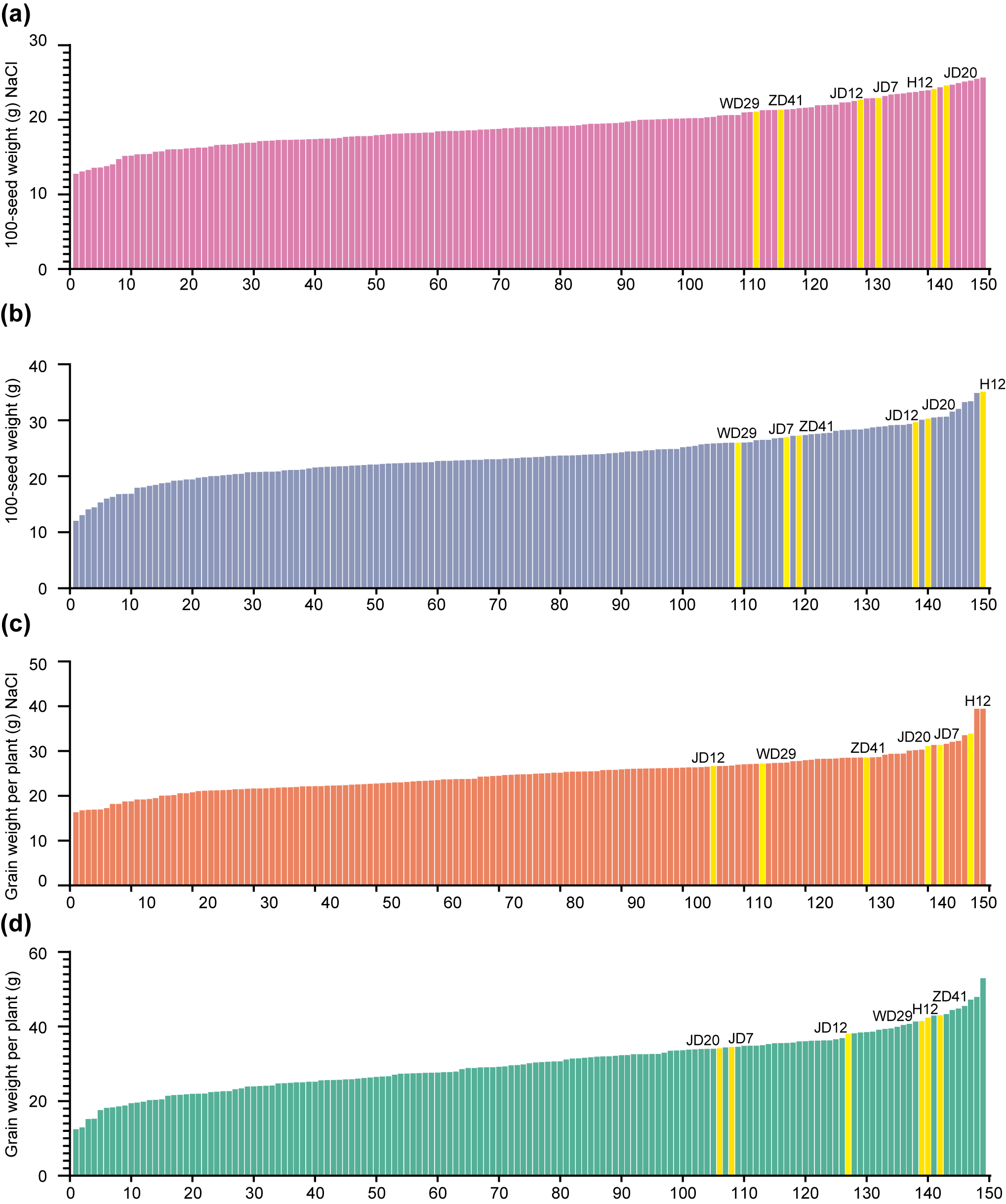

Supplement: Supplementary file 2 — Supporting Information [file ADVS-12-e09391-s003.zip › Figure-S11.tif]

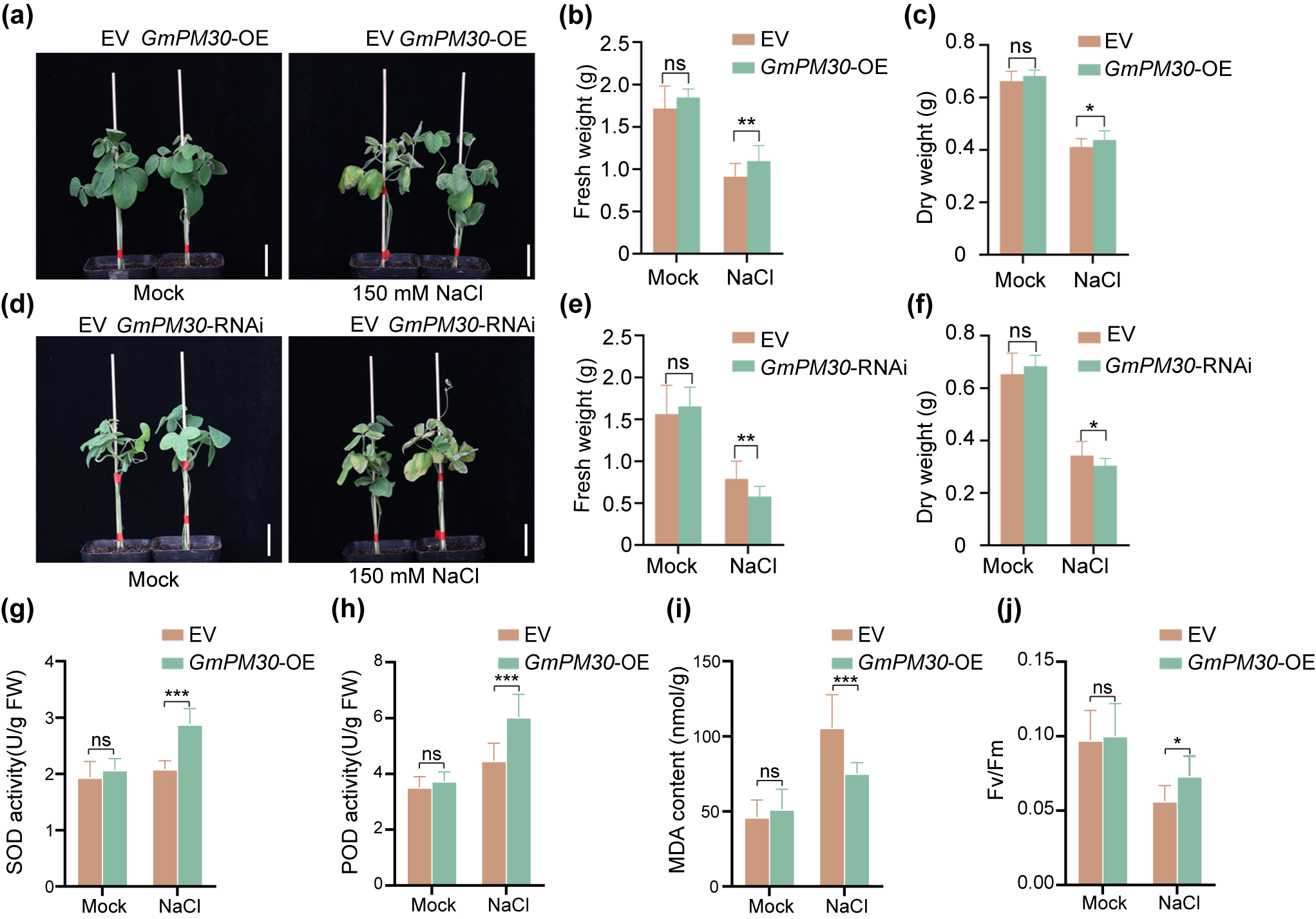

Supplement: Supplementary file 2 — Supporting Information [file ADVS-12-e09391-s003.zip › Figure-S2.tif]

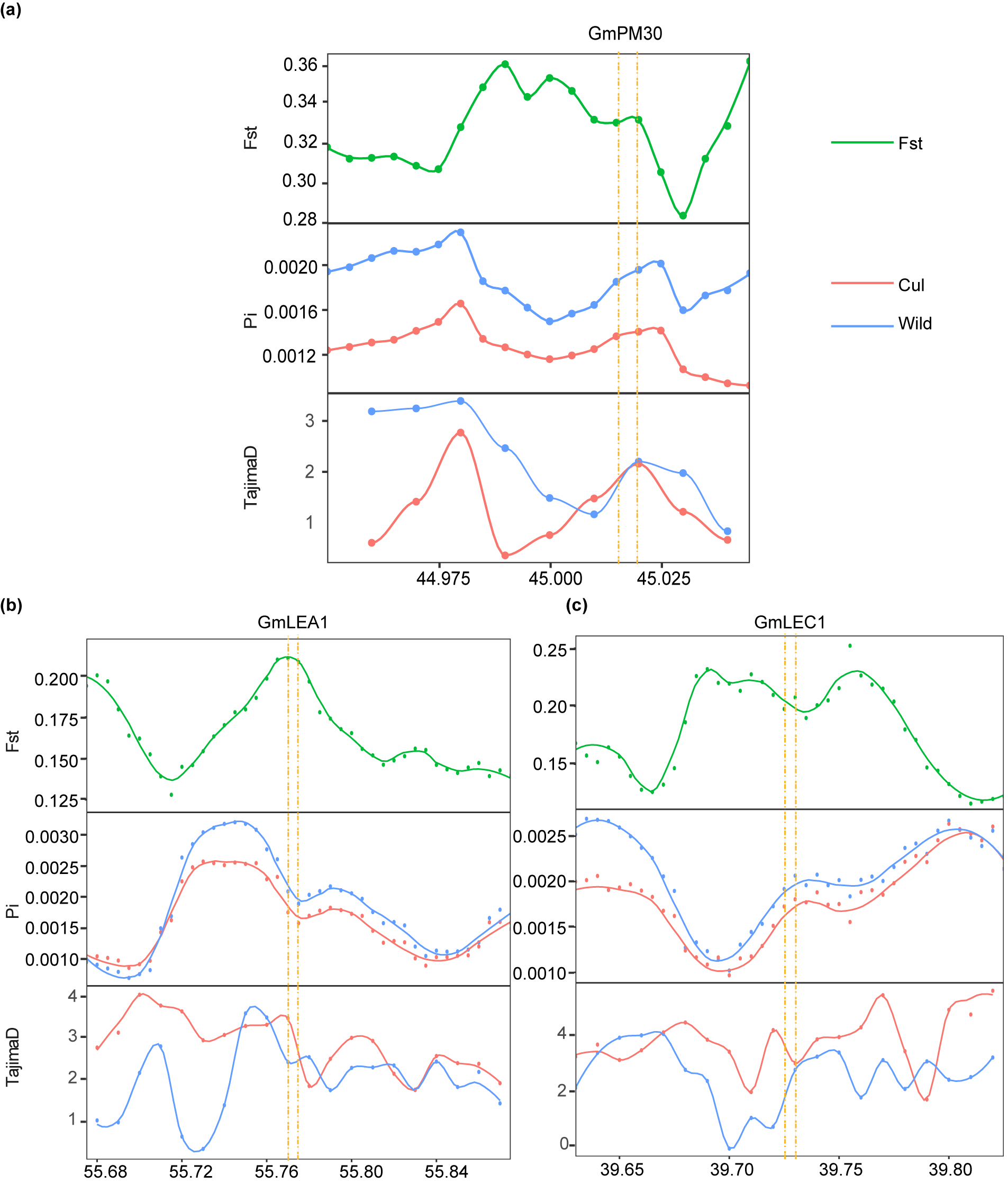

Supplement: Supplementary file 2 — Supporting Information [file ADVS-12-e09391-s003.zip › Figure-S3.tif]

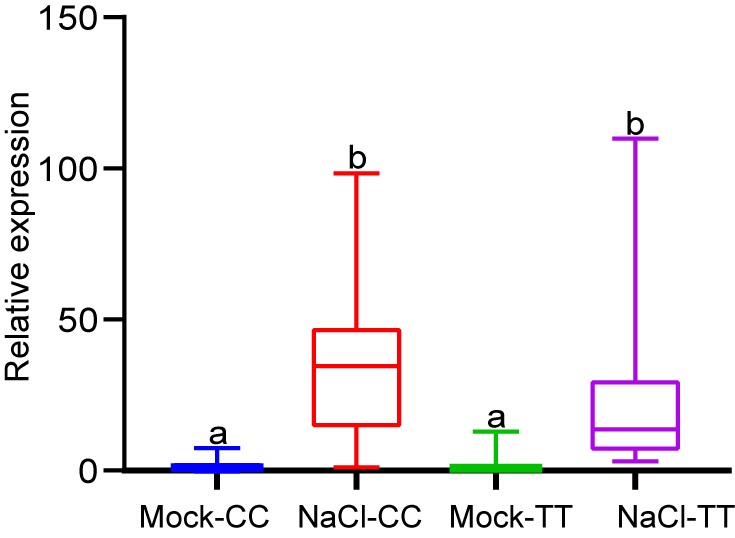

Supplement: Supplementary file 2 — Supporting Information [file ADVS-12-e09391-s003.zip › Figure-S4.tif]

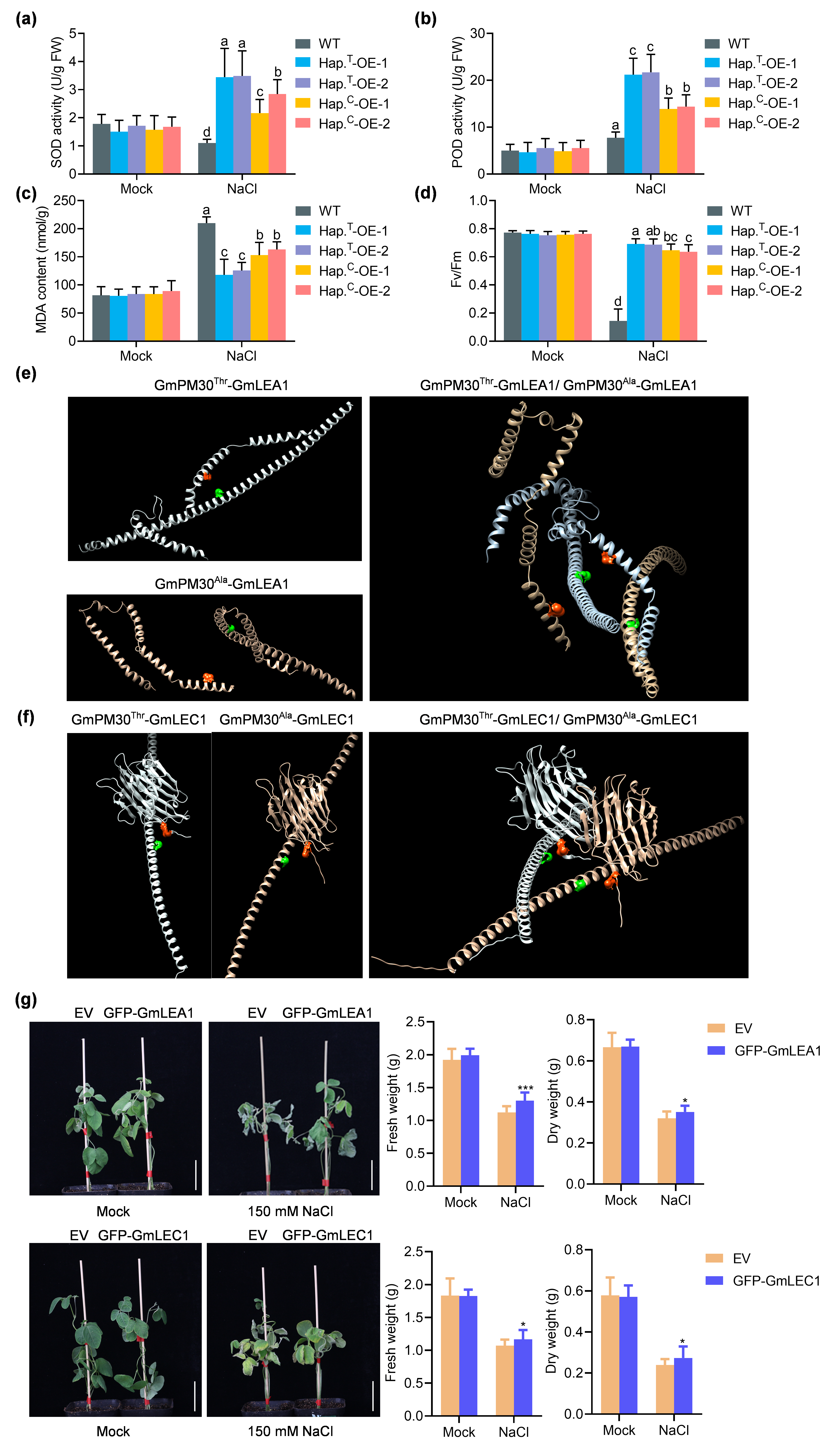

Supplement: Supplementary file 2 — Supporting Information [file ADVS-12-e09391-s003.zip › Figure-S5.tif]

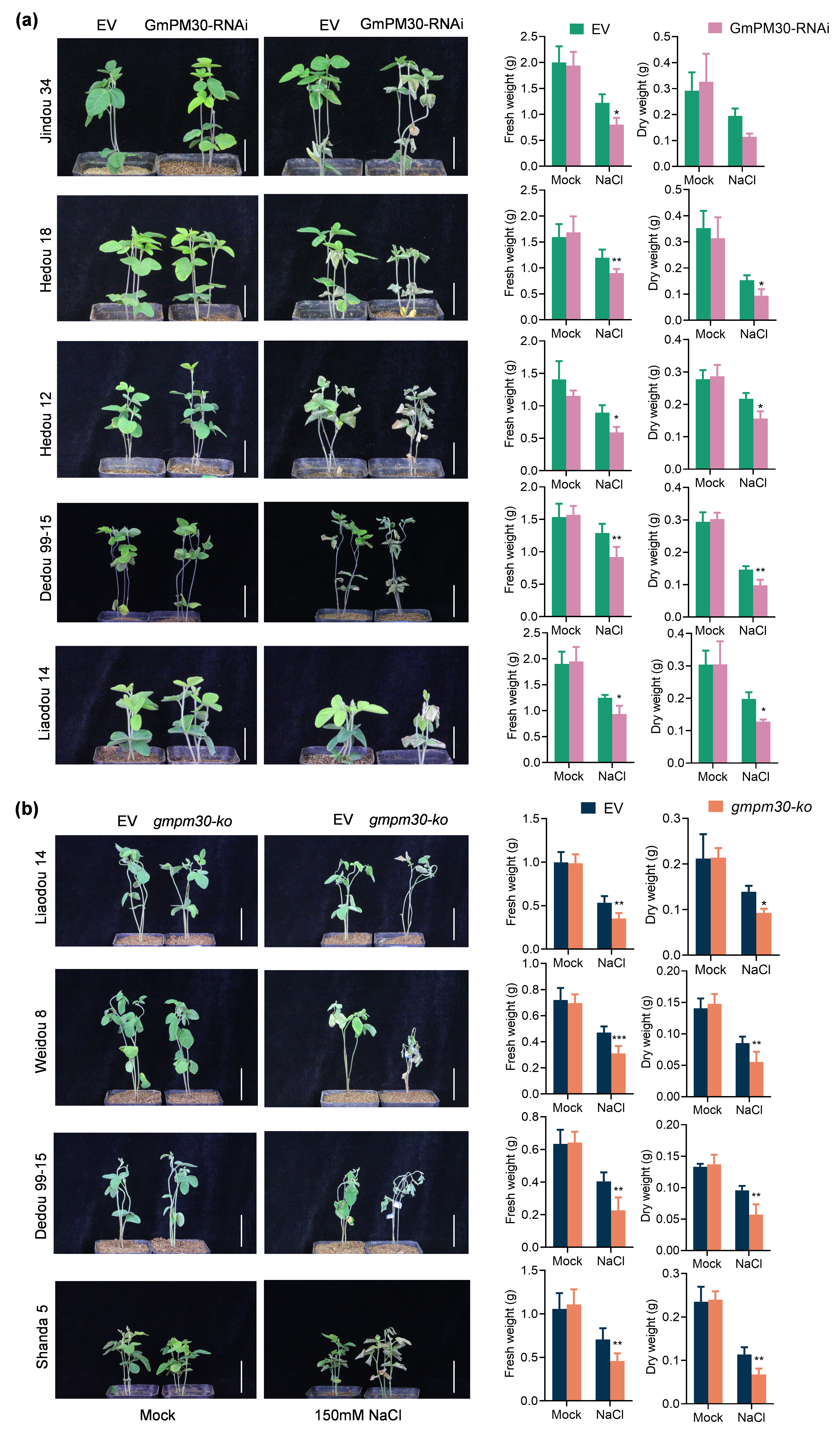

Supplement: Supplementary file 2 — Supporting Information [file ADVS-12-e09391-s003.zip › Figure-S6.tif]

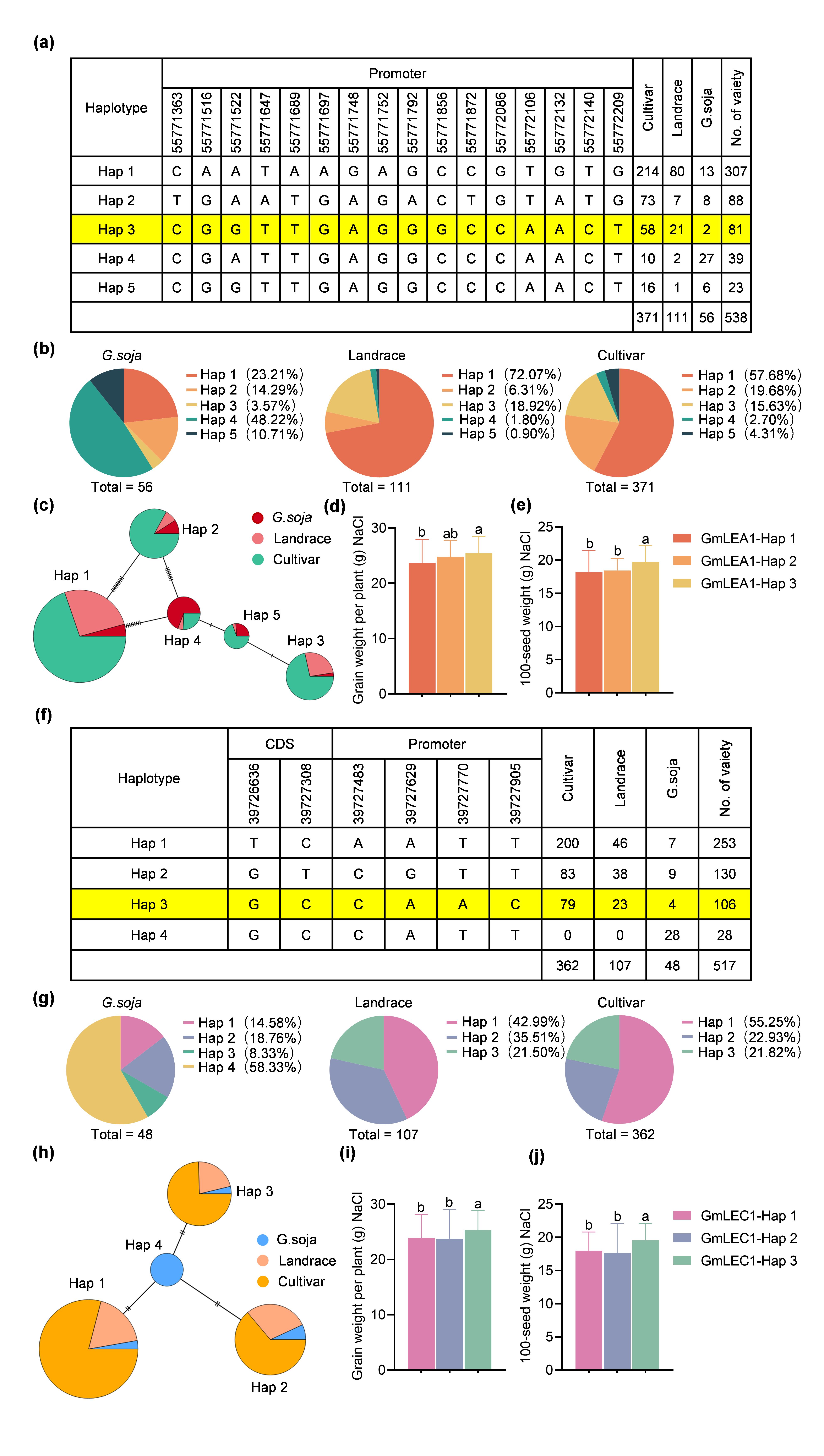

Supplement: Supplementary file 2 — Supporting Information [file ADVS-12-e09391-s003.zip › Figure-S7.tif]

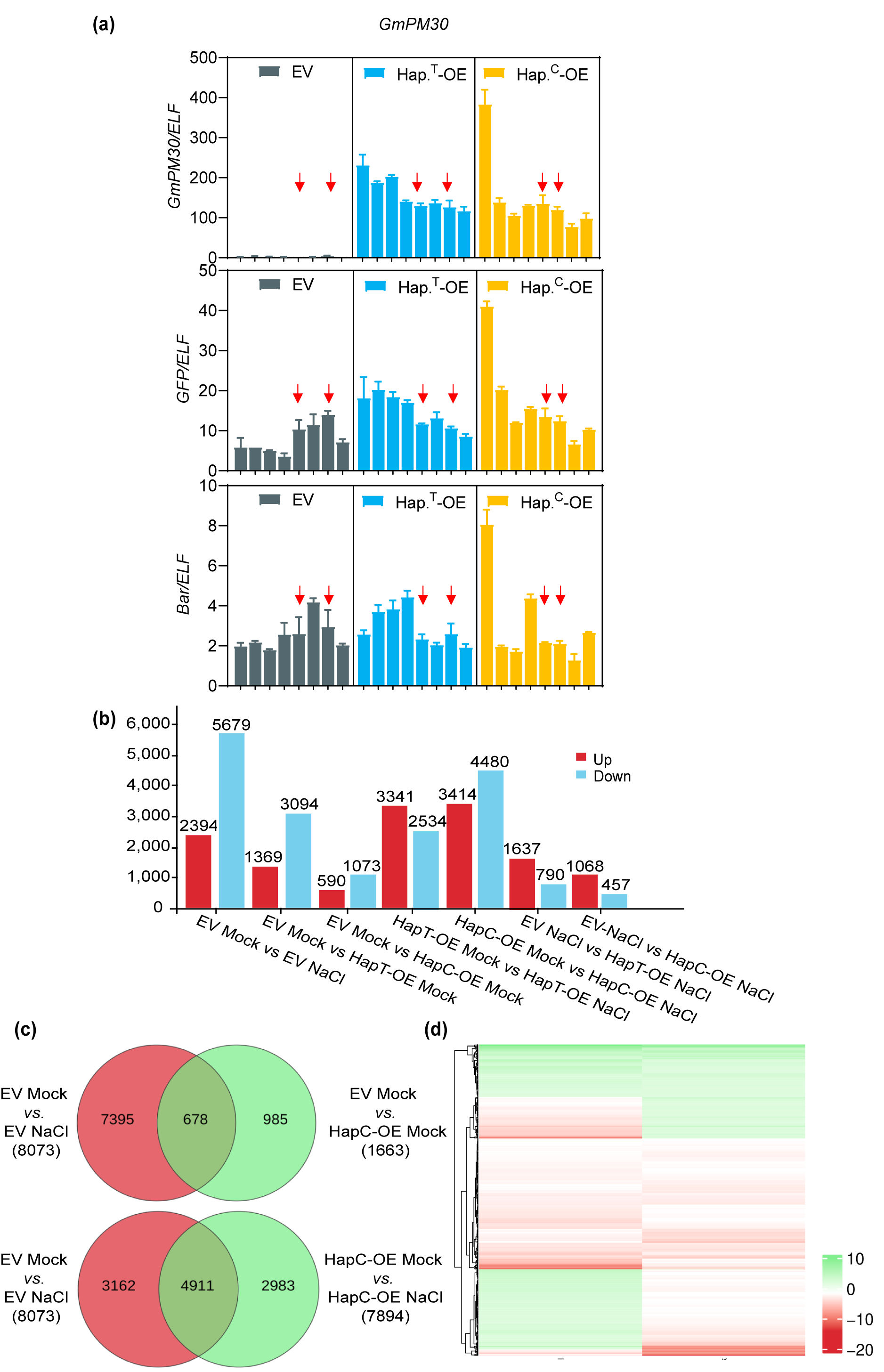

Supplement: Supplementary file 2 — Supporting Information [file ADVS-12-e09391-s003.zip › Figure-S8.tif]

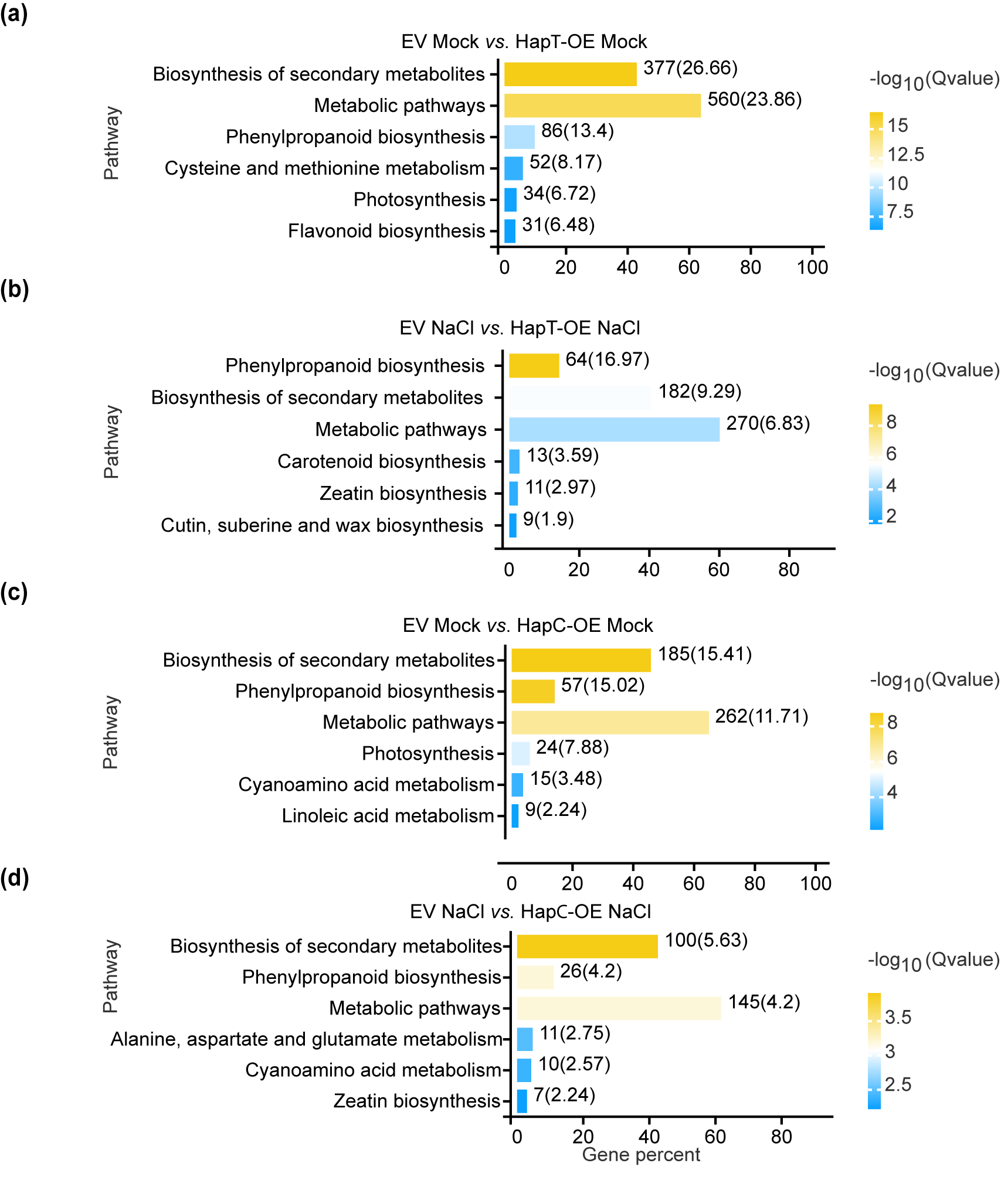

Supplement: Supplementary file 2 — Supporting Information [file ADVS-12-e09391-s003.zip › Figure-S9.tif]
